# Proteomes and ubiquitylomes reveal the regulation mechanism of cold tolerance mediated by *OsGRF4* in rice

**DOI:** 10.3389/fpls.2025.1531399

**Published:** 2025-03-21

**Authors:** Li Zhang, Renyan Huang, Donghai Mao, Jia Zeng, Pengpeng Fang, Qiang He, Fu Shu, Huafeng Deng, Wuhan Zhang, Pingyong Sun

**Affiliations:** ^1^ State Key Laboratory of Hybrid Rice, Hunan Hybrid Rice Research Center, Hunan Academy of Agricultural Sciences, Changsha, China; ^2^ Hunan Institute of Nuclear Agriculture Sciences and Chinese Herbal Medicines, Changsha, China; ^3^ Hunan Plant Protection Institute, Hunan Academy of Agricultural Science, Changsha, China; ^4^ Key Laboratory of Agro-Ecological Processes in Subtropical Region, Institute of Subtropical Agriculture, Chinese Academy of Science, Changsha, China; ^5^ Longping Branch, College of Biology, Hunan University, Changsha, China

**Keywords:** *Oryza sativa*, cold tolerance, OsGRF4 gene, proteomic analysis, ubiquitination modification

## Abstract

Low temperature is one of the major abiotic stresses that severely restrict the development of rice. It has been demonstrated previously that *OsGRF4* enhances cold tolerance in rice, the molecular mechanism of which remains unknown. This study employed a combination of proteome and ubiquitylome approaches to analyze *OsGRF4* mediated chilling between the overexpression line (OX) and wild type (CK). Proteome results showed that 6,157 proteins were identified and 5,045 proteins were quantified after 24-h cold treatment. A total of 59 proteins were upregulated and 63 proteins were downregulated in the OX24 vs. OX0 group; 27 proteins were upregulated and 34 proteins were downregulated in OX24 vs. CK24. Finally, 3,789 ubiquitination modification sites were located on 1,846 proteins, of which 2,695 sites of 1,376 proteins contained quantitative information. However, 178 sites in 131 proteins were quantified as upregulated and 92 sites in 72 proteins were quantified as downregulated differentially ubiquitin-modified proteins (DUMPs) in OX24 vs. OX0. To the contrary, 82 sites in 71 proteins were identified as upregulated and 13 sites in 12 proteins were identified as downregulated DUMPs in CK24 vs. OX24. The results suggested that global ubiquitination levels increase during cold tolerance in rice. In total, 76 differentially abundant proteins and 101 DUMPs were co-localized within 50 cold or stress tolerance Quantitative Trait Locis (QTLs). The combined analysis of proteomics and ubiquitination omics found that five proteins demonstrated opposing changes in protein and ubiquitination; the protein Q6ZH84 (Os02g0593700) was an upregulated differentially abundant protein (DAP) but was a downregulated DUMP in OX24 vs. OX0, which is a homologous gene of *NBR1* that regulated cold tolerance. Os02g0593700 should upregulate protein expression by reducing ubiquitination modification, thus affecting cold tolerance. The enrichment pathway shows that *OsGRF4* plays an important role in rice cold tolerance by ubiquitination through glutathione metabolism and arachidonic acid metabolism. The research provides a new perspective on the molecular mechanism of cold tolerance regulated by *OsGRF4*.

## 1 Introduction

Rice is one of the major food crops in the world, which is mainly cultivated in tropical and subtropical climate areas. Rice is sensitive to low-temperature climates, and the chilling injury seriously restricts its planting range, yield, and quality. With the increasing population pressure in the world, rice planting has been expanded to high-altitude latitudes, and cold damage has become increasingly frequent. Cloning cold tolerance genes and analyzing their molecular mechanism will lay a foundation for further study on rice adaptability to temperature (Chen et al., 2022). At present, the most thoroughly studied molecular mechanism of cold tolerance is the transcription factor *CBF*/*DREB1*-dependent response signaling pathway in *Arabidopsis thaliana*. *OsDREB1*, a *DREB1*/*CBF* homologous gene in Arabidopsis, positively regulates cold tolerance in rice (Ito et al., 2006). Because the promoters of these genes all have DRE *cis*-element and are regulated by *CBF*, they are also collectively called *CBF* regulon (Chinnusamy et al., 2007; Chen et al., 2013; Zhao et al., 2016; Corrales et al., 2017). *CTB1*, a key gene for cold tolerance at the booting stage, encodes an F-box protein (Saito et al., 2010). *LTG1* encodes a casein kinase I, which affects auxin transport, synthesis, and signal transduction and positively regulates the low-temperature tolerance of rice at the seedling stage (Lu et al., 2014). *COLD1*, a G protein regulator, positively regulates the cold tolerance of rice in the vegetative growth stage. It interacts with RGA1, a subunit of G protein, promotes calcium influx, and enhances GTP enzyme activity of G protein, thus enhancing the cold tolerance of rice (Ma et al., 2015). *HAN1* encodes a monooxygenase, which negatively regulates cold tolerance in rice. During the ecotype differentiation of indica rice and japonica rice, a specific allele from temperate japonica rice obtained a MYB *cis*-element in the HAN1 promoter, which enhanced the cold resistance of japonica rice at the seedling stage (Mao et al., 2019).

With the rapid development of sequencing technology, proteomics and modificomics analyses have become the common technical means to study the molecular mechanism of plant stress (Vu et al., 2018; Kosová et al., 2021). New isobaric tag for relative and absolute quantification (iTRAQ) and tandem mass tag (TMT) have been used for studying proteomics in response to cold stress in rice with relatively high efficiency and reproducibility. Studies of rice seedlings or germinated embryos and coleoptiles have shown that hundreds of proteins may play important roles in regulating cold tolerance and its natural variation among different germplasm (Neilson et al., 2011; Lee et al., 2015; Wang et al., 2018). Xie et al. reported the first rice ubiquitome and identified 861 di-Gly-Lys-containing peptides in 464 proteins in rice leaf cells by combining highly sensitive purification and high-resolution liquid chromatography–tandem mass spectrometry (LC-MS/MS) (Xie et al., 2015). Now, the modification omics analysis of the target gene has been carried out in rice gradually, and osmapk3-mediated phosphorylation inhibits ubiquitination of OsICE1, which indicates an interaction between post-transcriptional regulations (Zhang et al., 2017). In addition, RING E3 ubiquitin ligase encoded by *OsDIRP1* has been identified as a positive regulator of cold tolerance at the seedling stage, and its molecular basis needs further exploration (Cui et al., 2018). *OsPUS1* can directly bind to chloroplast rRNA precursors and catalyze pseudouridine modification of chloroplast rRNA. Combined transcriptome and translation group analysis demonstrated the key function of pseudouridine modification in chloroplast ribosome biosynthesis and cryogenic response in rice (Wang et al., 2022). Chilling-induced phosphorylation of *IPA1* by *OsSAPK6*, and *OsSAPK6* acts upstream of *IPA1* and positively regulates cold tolerance of rice through the *OsCBF*-dependent pathway (Jia et al., 2022).

A growth-regulating factor (GRF) is a plant-specific TF family, which is highly conserved in higher plants. There are 12 *GRF* members in rice. *OsGRF4* was first located in the 33.2-kb interval of the second chromosome and successfully cloned (Zhang et al., 2013). *OsGRF4* regulates grain length, grain width, 1000-grain weight, and yield of rice and improves nitrogen use efficiency (Hu et al., 2015; Che et al., 2015; Sun et al., 2016; Li et al., 2018; Chen et al., 2019). A study found that *OsGRF4* may participate in abiotic stress (Zhang et al., 2018). Through low-temperature stress treatment, the authors found that increasing the expression of *OsGRF4* can significantly improve the cold tolerance of rice seedlings. However, the molecular mechanism of *OsGRF4* controlling cold resistance is unknown. Proteomics and modificomics were applied to analyze the regulation mechanism of *OsGRF4* involving low-temperature stress. The results will enrich the theoretical basis of the molecular mechanism of plant cold tolerance and provide new gene resources for breeding cold-tolerant rice varieties.

## 2 Materials and methods

### 2.1 Rice materials

The plants of near-isogenic line (NIL) *Osgrf4* (CK) and the plants of overexpression of *OsGRF4* in NIL *Osgrf4* background (OX) were obtained from our research lab (Sun et al., 2016). The plants of CK and OX in the seedling stage were subjected to 4°C for 0 h (CK0 and OX0), 6 h (CK6 and OX6), and 24 h (CK24 and OX24). Three biological replicates of each seedling leaf were collected for proteomics and modificomics analyses by the PTM BIO Company, Hangzhou, China.

### 2.2 Protein extraction and trypsin digestion

The sample was ground under liquid nitrogen into cell powder and then transferred to a 5-mL centrifuge tube. After that, four volumes of lysis buffer (8 M urea, 1% Protease Inhibitor Cocktail) were added to the cell powder, followed by sonication three times on ice using a high-intensity ultrasonic processor (Scientz, Ningbo, China). [Note: For post-translational modification (PTM) experiments, inhibitors were also added to the lysis buffer, e.g., 3 μM Trichostatin A (TSA) and 50 mM nicotinamide (NAM) for acetylation.] The remaining debris was removed by centrifugation at 12,000 *g* and 4°C for 10 min. Finally, the supernatant was collected, and the protein concentration was determined using a bicinchoninic acid (BCA) kit according to the manufacturer’s instructions. For digestion, the protein solution was reduced with 5 mM dithiothreitol for 30 min at 56°C and alkylated with 11 mM iodoacetamide for 15 min at room temperature in darkness. The protein sample was then diluted by adding 100 mM Tetraethylammonium bromide (TEAB) to urea concentration at less than 2 M. Finally, trypsin was added at 1:50 trypsin-to-protein mass ratio for the first digestion overnight and 1:100 trypsin-to-protein mass ratio for the second 4-h digestion. The reagents used in this experiment were from the PTM BIO Company, Hangzhou, China.

### 2.3 Affinity enrichment

Pan antibody-based PTM enrichment: To enrich modified peptides, tryptic peptides dissolved in N-ethylmaleimide (NEM), Tris-HCl, EDTA and NaCl (NETN) buffer (100 mM NaCl, 1 mM Ethylene Diamine Tetraacetic Acid (EDTA), 50 mM Tris-HCl, and 0.5% NP-40, pH 8.0) were incubated with pre-washed antibody beads (Lot number 001, PTM BIO) at 4°C overnight with gentle shaking. Then, the beads were washed four times with NETN buffer and twice with H_2_O. The bound peptides were eluted from the beads with 0.1% trifluoroacetic acid. Finally, the eluted fractions were combined and vacuum-dried. For LC-MS/MS analysis, the resulting peptides were desalted with C18 ZipTips (Millipore, Billerica, MA, USA) according to the manufacturer’s instructions. The reagents used in this experiment were from the PTM BIO Company, Hangzhou, China.

### 2.4 LC-MS/MS analysis

The tryptic peptides were dissolved in 0.1% formic acid (solvent A) and directly loaded onto a homemade reversed-phase analytical column (15-cm length, 75 μm i.d.). The gradient was composed of an increase from 6% to 23% solvent B (0.1% formic acid in 98% acetonitrile) over 26 min, 23% to 35% in 8 min and climbing to 80% in 3 min then holding at 80% for the last 3 min, all at a constant flow rate of 400 nL/min on an EASY-nLC 1000 Ultra Performance Liquid Chromatography (UPLC) system. The peptides were subjected to an Nitrogen soluble index (NSI) source followed by tandem mass spectrometry (MS/MS) in Q Exactive™ Plus (Thermo, Waltham, MA, USA) coupled online to the UPLC. The electrospray voltage applied was 2.0 kV. The m/z scan range was 350 to 1,800 for a full scan, and intact peptides were detected in the Orbitrap at a resolution of 70,000. Peptides were then selected for MS/MS using Normalized Collision Energy (NCE) setting at 28, and the fragments were detected in the Orbitrap at a resolution of 17,500. A data-dependent procedure alternated between one MS scan, followed by 20 MS/MS scans with 15.0-s dynamic exclusion. Automatic gain control (AGC) was set as 5E4. The fixed first mass was set as 100 m/z. The reagents used in this experiment were from the PTM BIO Company, Hangzhou, China.

### 2.5 Database search

The resulting MS/MS data were processed using the MaxQuant search engine (v.1.5.2.8). Tandem mass spectra were searched against the human UniProt database concatenated with the reverse decoy database. Trypsin/P was specified as a cleavage enzyme, allowing up to four missing cleavages. The mass tolerance for precursor ions was set as 20 ppm in the first search and 5 ppm in the main search, and the mass tolerance for fragment ions was set as 0.02 Da. Carbamidomethyl on Cys was specified as a fixed modification, and acetylation modification and oxidation on Met were specified as variable modifications. False Discovery Rate (FDR) was adjusted to <1%, and the minimum score for modified peptides was set as >40.

### 2.6 Bioinformatics methods

Gene Ontology (GO) annotation proteome was derived from the UniProt-GOA database (http://www.ebi.ac.uk/GOA/). First, the identified protein ID was converted to UniProt ID and then mapped to GO IDs by protein ID. If some identified proteins were not annotated by the UniProt-GOA database, the InterProScan v.5.14-53.0 (http://www.ebi.ac.uk/interpro/) software was used to annotate the protein’s GO function based on protein sequence alignment method. Then, proteins were classified by Gene Ontology annotation based on three categories: biological process, cellular component, and molecular function. The GO with a corrected *p*-value < 0.05 was considered significant.

Identified protein domain functional descriptions were annotated by InterProScan (a sequence analysis application) based on the protein sequence alignment method, the InterPro (http://www.ebi.ac.uk/interpro/) domain database was used, and protein domains with a corrected *p*-value < 0.05 were considered significant. The Kyoto Encyclopedia of Genes and Genomes (KEGG) database (KAAS v.2.0 http://www.genome.jp/kaas-bin/kaas_main; KEGG Mapper v2.5 http://www.kegg.jp/kegg/mapper.html) was used to annotate the protein pathway. The pathway with a corrected *p*-value < 0.05 was considered significant.

WoLF PSORT, a subcellular localization prediction software, was used to predict subcellular localization. WoLF PSORT v.0.2 (http://www.genscript.com/psort/wolf_psort.html) is an updated version of PSORT/PSORT II for the prediction of eukaryotic sequences. Especially for prokaryotic species, subcellular localization prediction software CELLO v.2.5 (http://cello.life.nctu.edu.tw/) was used.

Software MoMo v5.0.2 (motif-x algorithm) (http://meme-suite.org/tools/momo) was used to analyze the model of sequences constituted with amino acids in specific positions of modify-21-mers (10 amino acids upstream and downstream of the site, but phosphorylation with modify-13-mers of six amino acids upstream and downstream of the site) in all protein sequences.

Enrichment-based clustering: For further hierarchical clustering based on differentially modified protein functional classification (such as GO, Domain, Pathway, and Complex). First, all the categories obtained were collated after enrichment along with their *p*-values and then filtered for those categories that were at least enriched in one of the clusters with *p*-value <0.05. This filtered *p*-value matrix was transformed by the function x = −log10 (*p*-value). Finally, these x values were z-transformed for each functional category. These z scores were then clustered by one-way hierarchical clustering (Euclidean distance and average linkage clustering) in Genesis. Cluster membership was visualized by a heat map using the “heatmap.2” function from the “gplots” R package v.2.0.3 (https://cran.r-project.org/web/packages/cluster/).

Protein–protein interaction network: All differentially expressed modified protein database accessions or sequences were searched against the STRING database version 10.5 for protein–protein interactions. Only interactions between the proteins belonging to the searched data set were selected, thereby excluding external candidates. STRING defines a metric called “confidence score” to define interaction confidence; all interactions that had a confidence score ≥0.7 (high confidence) were fetched. Interaction network form STRING was visualized in the R package “networkD3” v.0.4 (https://cran.r-project.org/web/packages/networkD3/).

Parallel Reaction Monitoring (PRM) data analysis: The resulting MS data were processed using Skyline (v.3.6). Peptide settings: The enzyme was set as Trypsin [KR/P], and the max missed cleavage was set as 2. The peptide length was set as 8–25, variable modification was set as carbamidomethyl on Cys and oxidation on Met, and max variable modifications were set as 3. Transition settings: Precursor charges were set as 2 and 3; ion charges were set as 1 and 2; and ion types were set as b, y, and p. The product ions were set from ion 3 to the last ion, and the ion match tolerance was set as 0.02 Da.

## 3 Results

### 3.1 Cold treatment changes the proteome profile in rice leaves

**Figure 1:**
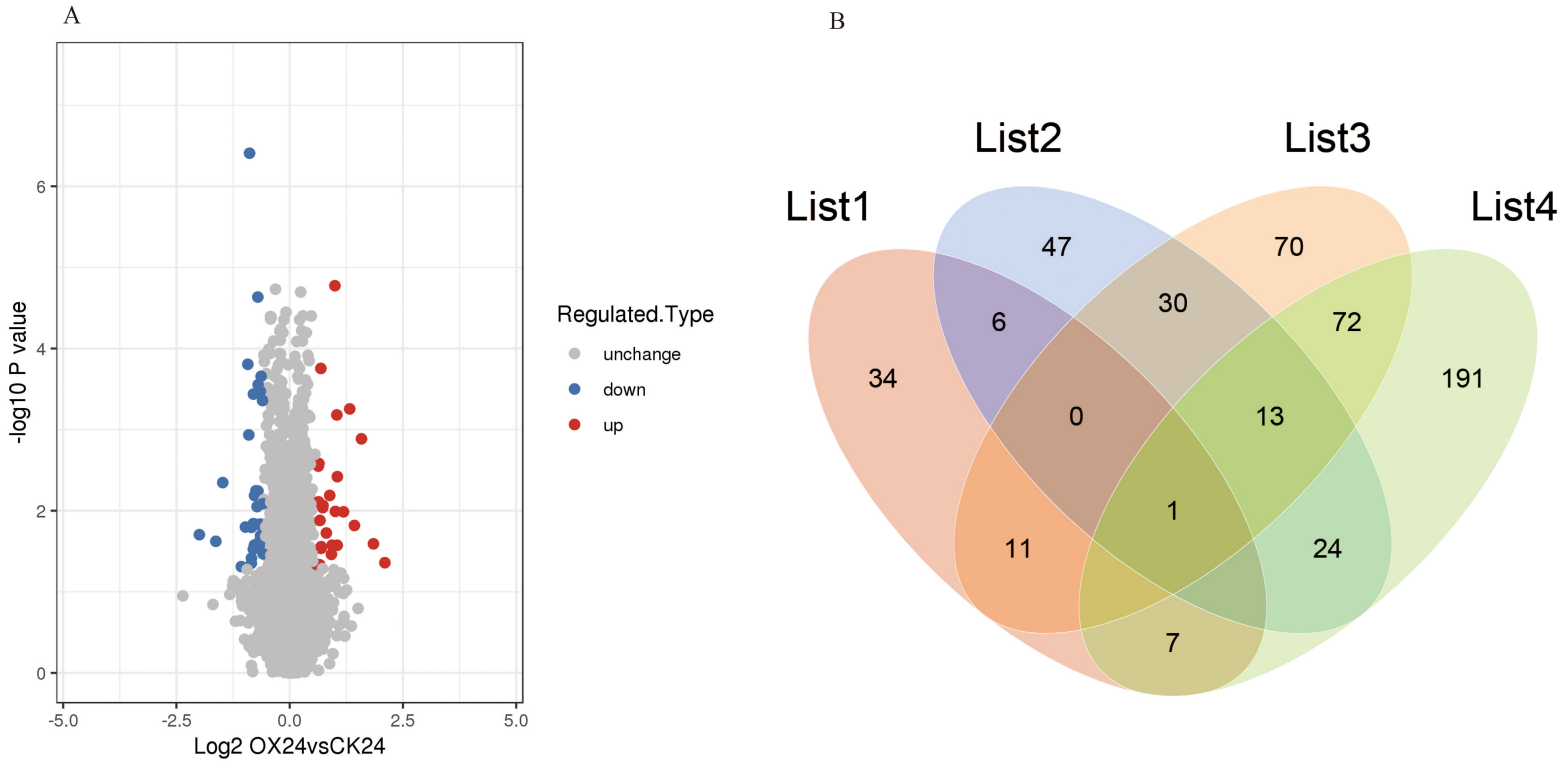
Volcano plot and Venn diagram of identified DAPs. **(A)** DAPs identified in OX24 vs. CK24. **(B)** List 1, OX24 vs. CK24; List 2, OX24 vs. OX0; List 3, CK24 vs. CK0; List 4, OX0 vs. CK0. DAPs, differentially abundant proteins.

To detect the whole proteome of four rice materials (CK0, CK24, OX0, and OX24) in response to cold, three biological replicates were analyzed for each treatment. In total, 6,157 (73.7%) protein groups based on 35,889 unique peptides were identified, among which 5,045 proteins were quantified with a high degree of repeatability (**Supplementary Table 1**). Principal component analysis (PCA) and relative standard deviation (RSD) were used to evaluate the repeatability of protein quantification. The results demonstrated that the three replicates of four rice materials were highly reproducible (**Supplementary Figures 1**, **2**). Based on two-way ANOVA followed by Student’s t-test analysis, differentially abundant proteins (DAPs) were identified, a total of 59 proteins were upregulated, and 63 proteins were downregulated (filtered using the criteria of *p* < 0.05 and a fold change >1.50 or <0.67) in the OX24 vs. OX0 group in response to cold (**Supplementary Tables 2**, **3**). A total of 27 proteins were upregulated and 34 proteins were downregulated in OX24 vs. CK24 (**Supplementary Table 4**; **Figure 1A**), in which 10 proteins were upregulated with fold change >2.0, and Os05g0392050 encoding E3 ubiquitin-protein ligase had the highest fold change (4.29). The Venn diagram demonstrated the specific and common DAPs identified in different comparison groups (**Figure 1B**); 34 and 47 specific DAPs were identified in OX24 vs. CK24 and OX24 vs. OX0, respectively. Six common DAPs (LOC_Os06g05520, LOC_Os04g45000, LOC_Os12g06780, LOC_Os02g17920, LOC_Os11g45990, and LOC_Os06g26234) were identified between OX24 vs. CK24 and OX24 vs. OX0 (**Supplementary Tables 3**, **4**); those specific and common DAPs may play an important role in cold resistance regulation.

GO enrichment analysis was performed to elucidate the functional differences of the DAPs identified in the OX24 vs. CK24 group. In the biological process classification, the majority of both upregulated and downregulated DAPs were involved in the metabolic process, cellular process, single-organism process, and response to stimulus (**Supplementary Figure 3**). In terms of the cellular component category, cell and organelle were the top two categories enriched by both upregulated and downregulated DAPs. In terms of molecular function, the top two classes were binding and catalytic activity. These results suggested that *OsGRF4* responded to cold treatment by simultaneously promoting and inhibiting the expression of specific proteins. In the subcellular localization analysis, the three largest categories were chloroplast, cytoplasm, and nucleus. The percentage of DAPs located in chloroplast was 45.08% and 45.90% in the groups OX24 vs. OX0 and OX24 vs. CK24, respectively, implying that proteins in chloroplast were the major members involved in cold response.

### 3.2 Protein ubiquitylation is involved in the regulation of cold resistance in rice

**Figure 2:**
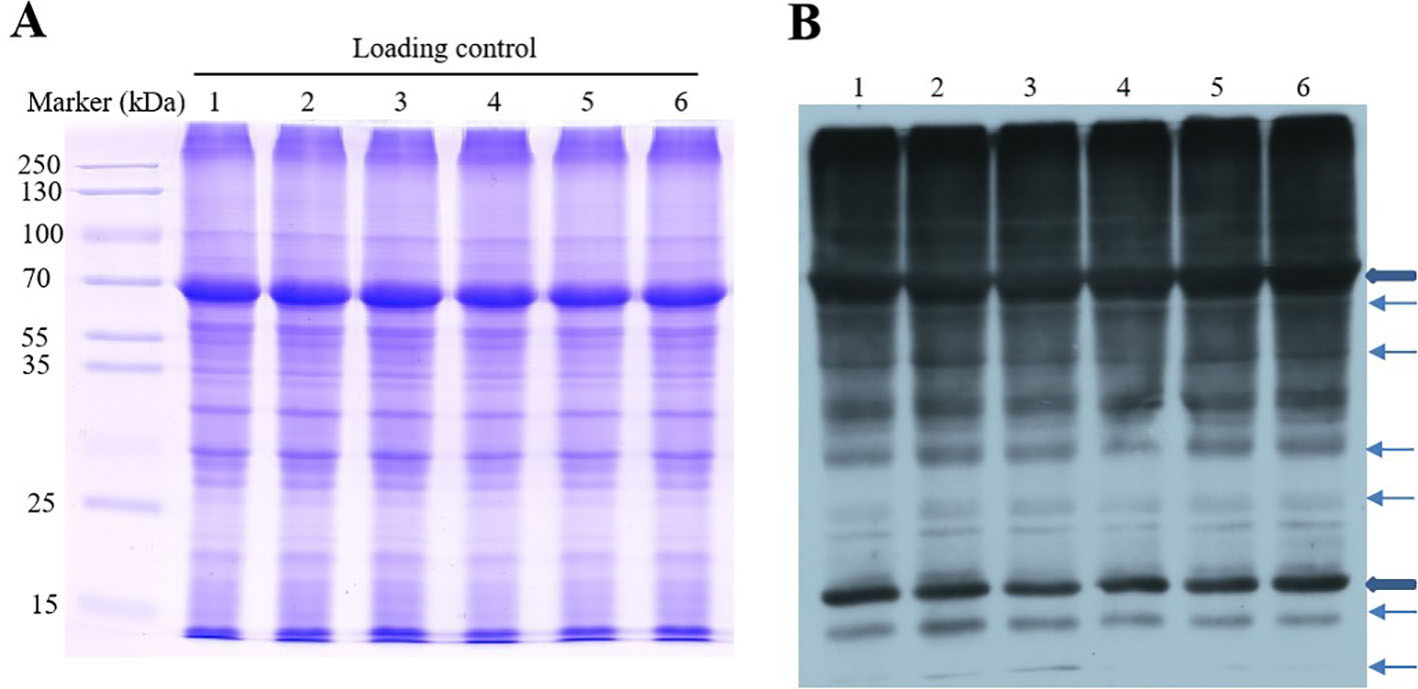
Identification of protein ubiquitylation after cold treatment. **(A)** Sodium Dodecyl Sulfate-Polyacrylamide Gel Electrophoresis (SDS–PAGE) gel stained with Coomassie brilliant blue as the loading control. **(B)** Western blotting with pan anti-ubiquitin antibody. 1, CK0h; 2, CK6h; 3, CK24h; 4, OX0h; 5, OX6h; 6, OX24h.

Ubiquitination plays an important role in protein degradation as an important PTM. In previous studies, the cold resistance of plants also was regulated by ubiquitination (Zhang et al., 2017). Therefore, to investigate whether *OsGRF4*-mediated cold resistance was involved in PTM, protein ubiquitylation dynamic was detected by immunoblot analysis with anti-ubiquitin antibody in rice at 0, 6, and 24 h after cold treatment. As shown in **Figure 2**, a significant ubiquitylation level is shown on the top of the gel (70 kDa), implying the promotion of poly-ubiquitylation. The protein ubiquitylation signals were observably changed from 0 to 24 h and achieved a high level at 24 h in *OsGRF4*-OX. The ubiquitylation signals of bands of 10, 15, and 70 kDa, etc., were significantly different between OX24 and CK24 or between OX24 and OX0 after cold treatment; hence, the ubiquitylomes of OX24, OX0, CK24, and CK0 were conducted in the following research. These results showed that the ubiquitylation modification may play an important role in *OsGRF4*-mediated cold resistance.

### 3.3 Identification of ubiquitylome profiling in rice after cold treatment

Label-free affinity enrichment and high-resolution liquid chromatography–mass spectrometry were combined to quantify protein ubiquitination in OX24, OX0, CK24, and CK0 after cold treatment. In total, after investigating three replicates for each material, 3,789 ubiquitination sites were identified on 1,846 proteins, of which 2,695 sites of 1,376 proteins (1.9 average sites per protein) with a high degree of reproducibility were accurately quantified (**Supplementary Table 5**, **Supplementary Figures 4**, **5**). The protein quantitative group was normalized to eliminate the influence of protein expression on the modified signal, and the data were used for subsequent bioinformatics analysis.

Based on two-way ANOVA followed by Student’s t-test analysis, differentially ubiquitin-modified proteins (DUMPs) were identified. Filtered using the criteria of *p* < 0.05 and a fold change >1.50 or <0.67, a total of 178 sites in 131 proteins were quantified as upregulated and 92 sites in 72 proteins were quantified as downregulated DUMPs in OX24 vs. OX0 in response to cold. To the contrary, 82 sites in 71 proteins were identified as upregulated and 13 sites in 12 proteins were identified as downregulated DUMPs in OX24 vs. CK24 (**Table 1**; **Supplementary Tables 6**, **7**). These results suggested that *OsGRF4*-mediated cold resistance greatly increased the level of ubiquitination.

**Table 1:** Differentially ubiquitin-modified sites (modified proteins) after normalized summary.

Compare group	Regulated type	Fold change > 1.2	Fold change > 1.3	Fold change > 1.5	Fold change > 2
CK24/CK0	Upregulated	133 (110)	118 (100)	90 (78)	44 (38)
	Downregulated	301 (208)	262 (193)	200 (155)	98 (87)
OX0/CK0	Upregulated	25 (20)	17 (14)	11 (9)	2 (1)
	Downregulated	258 (196)	204 (165)	121 (101)	32 (27)
OX24/CK24	Upregulated	154 (122)	124 (103)	82 (71)	33 (29)
	Downregulated	41 (39)	27 (25)	13 (12)	4 (4)
OX24/OX0	Upregulated	239 (177)	218 (161)	178 (131)	99 (80)
	Downregulated	146 (110)	133 (102)	92 (72)	40 (33)

### 3.4 GO category, subcellular localization, and Clusters of Orthologous Groups analysis of the DUMPs

**Figure 3:**
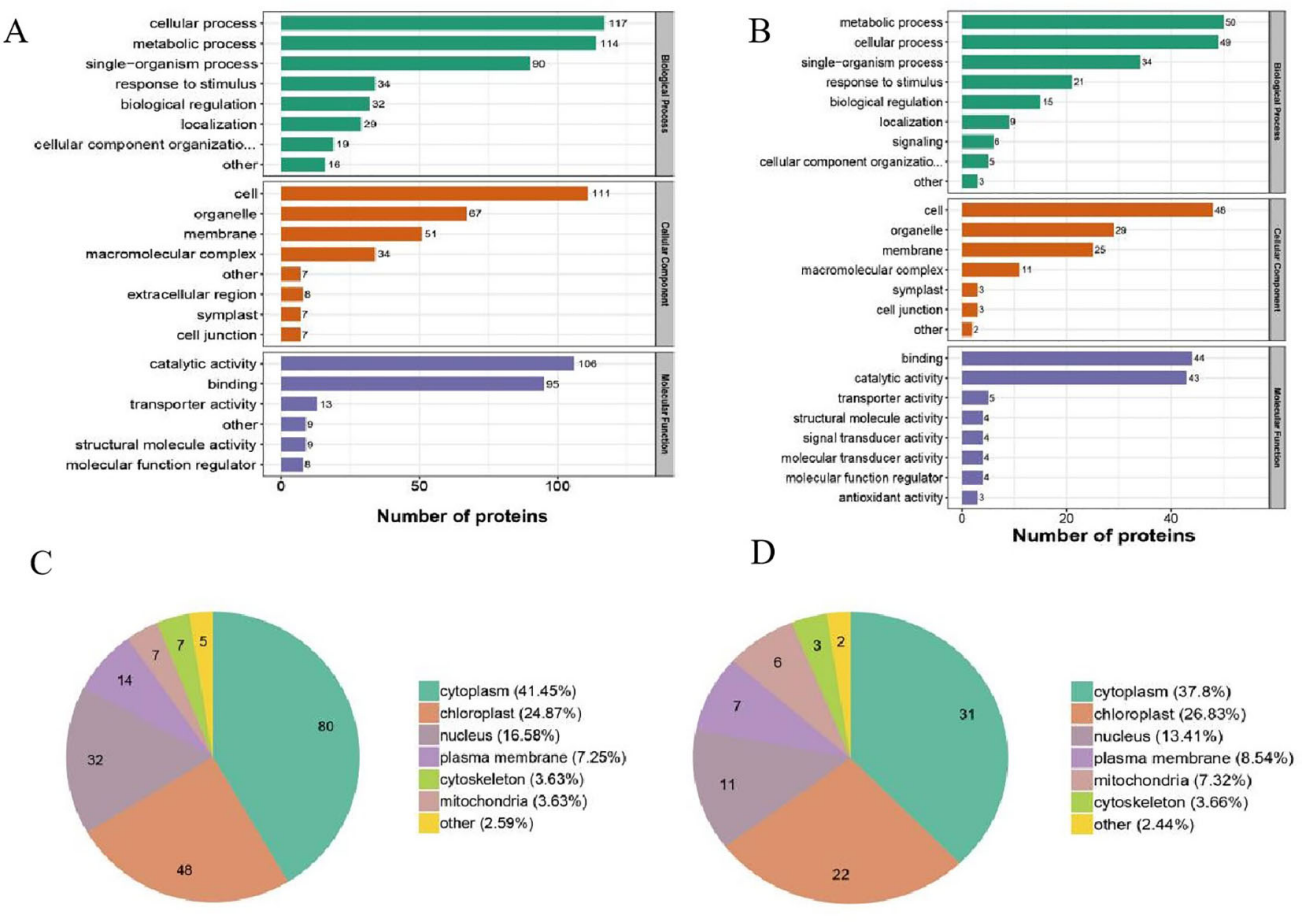
Functional enrichment and subcellular localization analyses of DUMPs. **(A, B)** GO-based enrichment analysis of DUMPs in OX24/OX0 group and OX24/CK24 group. **(C, D)** Subcellular localization of DUMPs in OX24/OX0 group and OX24/CK24 group. DUMPs, differentially ubiquitin-modified proteins; GO, Gene Ontology.

To better understand the potential functions of the DUMPs after cold treatment, a Gene Ontology functional annotation analysis was performed on all identified ubiquitinated proteins into three categories, biological process (BP), cellular component (CC), and molecular function (MF). The upregulated DUMPs of OX24/OX0 were enriched in 22 functional categories (eight BP, eight CC, and six MF), while the downregulated DUMPs were enriched in 24 functional categories (nine BP, nine CC, and six MF) (**Figure 3A**; **Supplementary Table 8**). In the BP category, the most enriched terms were “metabolic process” and “cellular process” in both upregulated and downregulated DUMPs; the “cell”, “organelle”, and “membrane” were significantly enriched in the CC category. In the MF category, the GO terms “catalytic activity” and “binding” showed the highest enrichment. The upregulated DUMPs of OX24/CK24 were enriched in 23 functional categories (nine BP, six CC, and eight MF), while downregulated DUMPs were enriched in 20 functional categories (six BP, eight CC, and six MF). The enriched terms in the three categories were similar to those in the OX24/OX0 comparison group, but the number of the DUMPs dramatically decreased in the OX24/CK24 group. There were 640 upregulated and 384 downregulated DUMPs in the OX24/OX0 group and only 365 upregulated and 75 downregulated DUMPs enriched in the OX24/CK24 group (**Figure 3B**; **Supplementary Table 8**). These results show that cold treatment likely promotes many metabolic processes while inhibiting certain biosynthetic processes, suggesting an intrinsic role in cold stress.

Post-translational modified proteins are transported to different organelles to perform their functions. In order to understand the function of DUMPs, we performed subcellular localization prediction of DUMPs in the OX24/OX0 group and OX24/CK24 group. The results showed that the DUMPs were mainly distributed in the cytoplasm (OX24/OX0 41.45%; OX24/CK24 37.8%), chloroplast (OX24/OX0 24.87%; OX24/CK24 26.83%), nucleus (OX24/OX0 16.58%; OX24/CK24 13.41%), and plasma membrane (OX24/OX0 7.25%; OX24/CK24 8.54%) (**Figures 3C,D**; **Supplementary Table 9**). We hypothesize that the DUMPs located in the cytoplasm and chloroplast are important participants in the process of cold tolerance.

The identified DUMPs were classified into 21 Clusters of Orthologous Groups (COG)/KOG categories in the comparison group OX24/OX0 and 15 COG/KOG categories in the comparison group OX24/CK24 (**Supplementary Figure 6**; **Supplementary Table 10**). The group “post-translational modification, protein turnover, chaperones” was the largest in both the OX24/OX0 comparison (33 DUMPs) and OX24/CK24 comparison (10 DUMPs), followed by the “translation, ribosomal structure and biogenesis” group (15 DUMPs) and “energy production and conversion” group (13 DUMPs), and the “translation, ribosomal structure and biogenesis” group (eight DUMPs) and “carbohydrate transport and metabolism” group (six DUMPs) in the OX24/OX0 and OX24/CK24 comparison groups, respectively.

### 3.5 KEGG pathway and protein domain enrichment analyses

**Figure 4:**
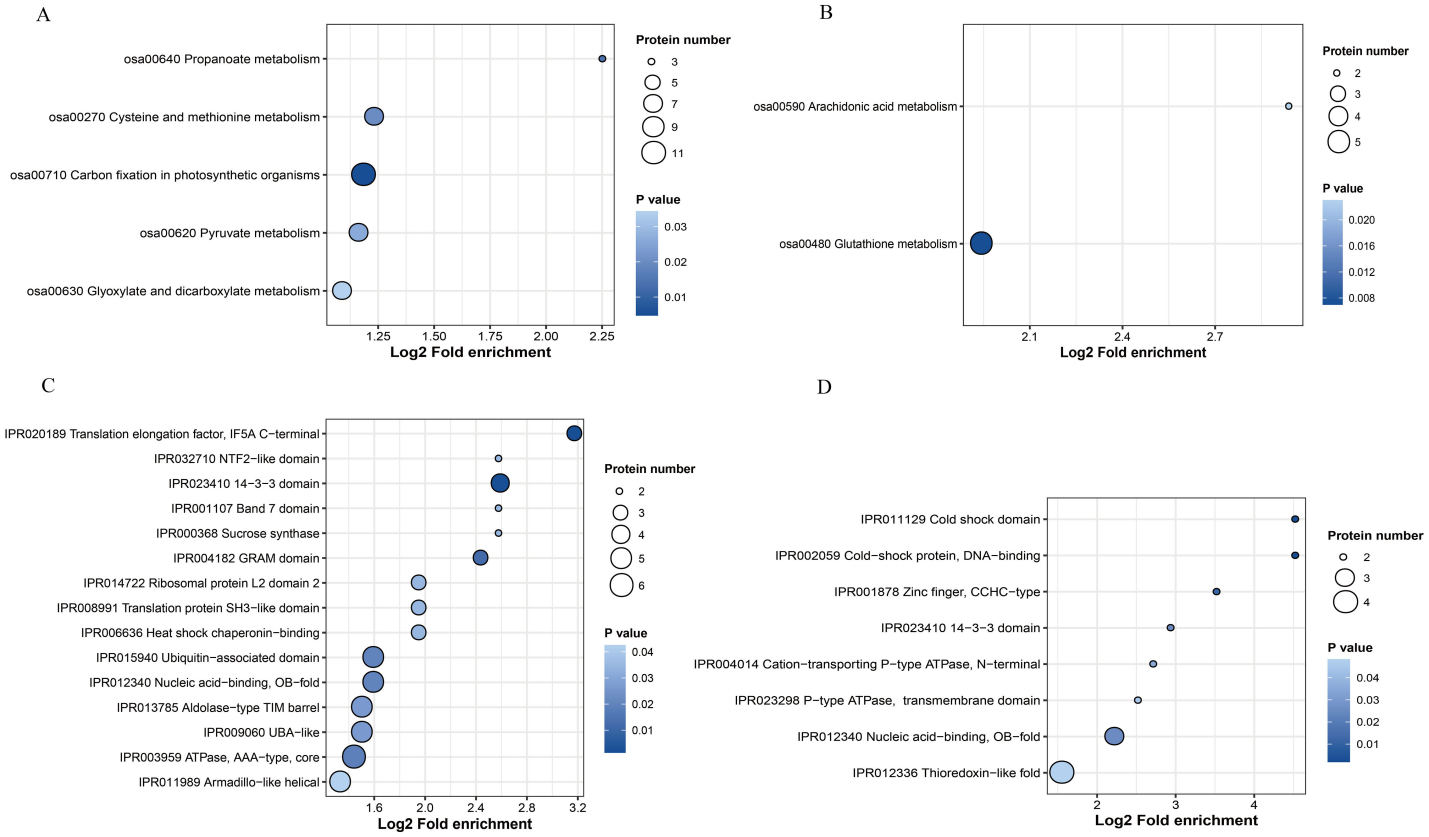
KEGG pathway and protein domain enrichment analyses. **(A, C)** OX24/OX0 and **(B, D)** OX24/CK24 comparison groups. KEGG, Kyoto Encyclopedia of Genes and Genomes.

To elucidate the functions of the proteins that underwent ubiquitination, we performed the KEGG pathway and protein domain analysis. In total, 35 and seven proteins were mapped in five and two KEGG pathways in the OX24/OX0 and OX24/CK24 comparison groups, respectively (**Figures 4A, B**). A number of vital pathways, including those related to propanoate metabolism, cysteine and methionine metabolism, pyruvate metabolism, and glutathione metabolism, were significantly enriched among DUMPs in the OX24/OX0 group. However, only glutathione metabolism and arachidonic acid metabolism were mapped in the OX24/CK24 group, and the glutathione metabolism was mapped in the two comparison groups. We also observed that 56 proteins were mapped in 15 domains in the OX24/OX0 group, such as translation elongation factor, 14-3-3 domain, and ubiquitin-associated domain; 19 proteins were mapped in eight domains in the OX24/CK24 group, and the cold shock protein, cold shock domain, and 14-3-3 domain were enriched in this comparison group (**Figures 4C, D**). These results suggested that ubiquitination may be highly associated with the amino acid metabolism pathway under cold stress in rice.

### 3.6 Motif analysis of ubiquitinated sites

**Figure 5:**
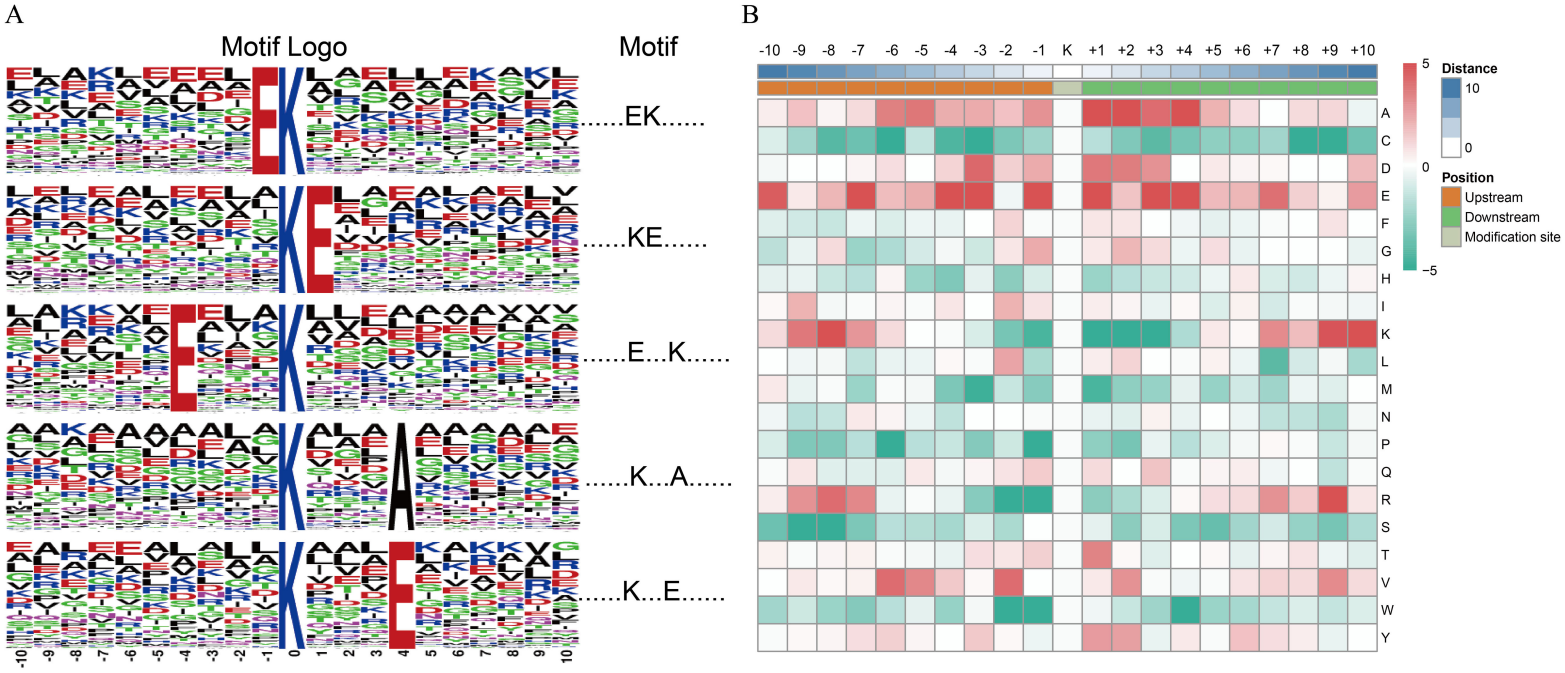
Motif analysis of all identified ubiquitination modification sites. **(A)** Ubiquitination motifs and the conservation of ubiquitination modification sites. The height of each letter corresponds to the frequency of that amino acid residue in that position. The central K refers to the ubiquitinated Lys. **(B)** Amino acid sequence properties of ubiquitylation sites. The heat map shows significant position-specific under-representation or over-representation of amino acids flanking the modification sites.

To understand the properties of the identified consensus, peptide motif(s) for the ubiquitination modification sites were extracted using the MOTIF-X software. In total, five ubiquitination modification motifs were defined on 1,376 (51%) unique sites. The five unique sites were designated………EK……….,……….KE………,….E … K……….,……….K … A……, and……….K … E……; they exhibited different abundances (indicates any amino acid) (**Figure 5A**). Among them, the motif………EK………. has been reported previously in rice (Xie et al., 2015). We surveyed the motifs and found that only two distinct residues were found upstream or downstream of the ubiquitinated Lys residues, and glutamic acid (E) and aspartic acid (D) were highly enriched around the ubiquitination modification sites. Alanine (A: neutral) was also overrepresented (*p* < 0.001) surrounding Kub sites (**Figure 5A**), whereas in rice, only A and E were observed surrounding ubiquitinated Lys residues (Xie et al., 2015). However, the basophilic residues histidine (H), arginine (R), methionine (M), proline (P), and lysine (K) were excluded from the adjacent positions (**Figure 5B**). These results show the differences in ubiquitinated Lys motifs between the normal and cold stress treatment in rice seedlings.

### 3.7 Protein-protein interaction (PPI) networks and metabolic pathway analysis

**Figure 6:**
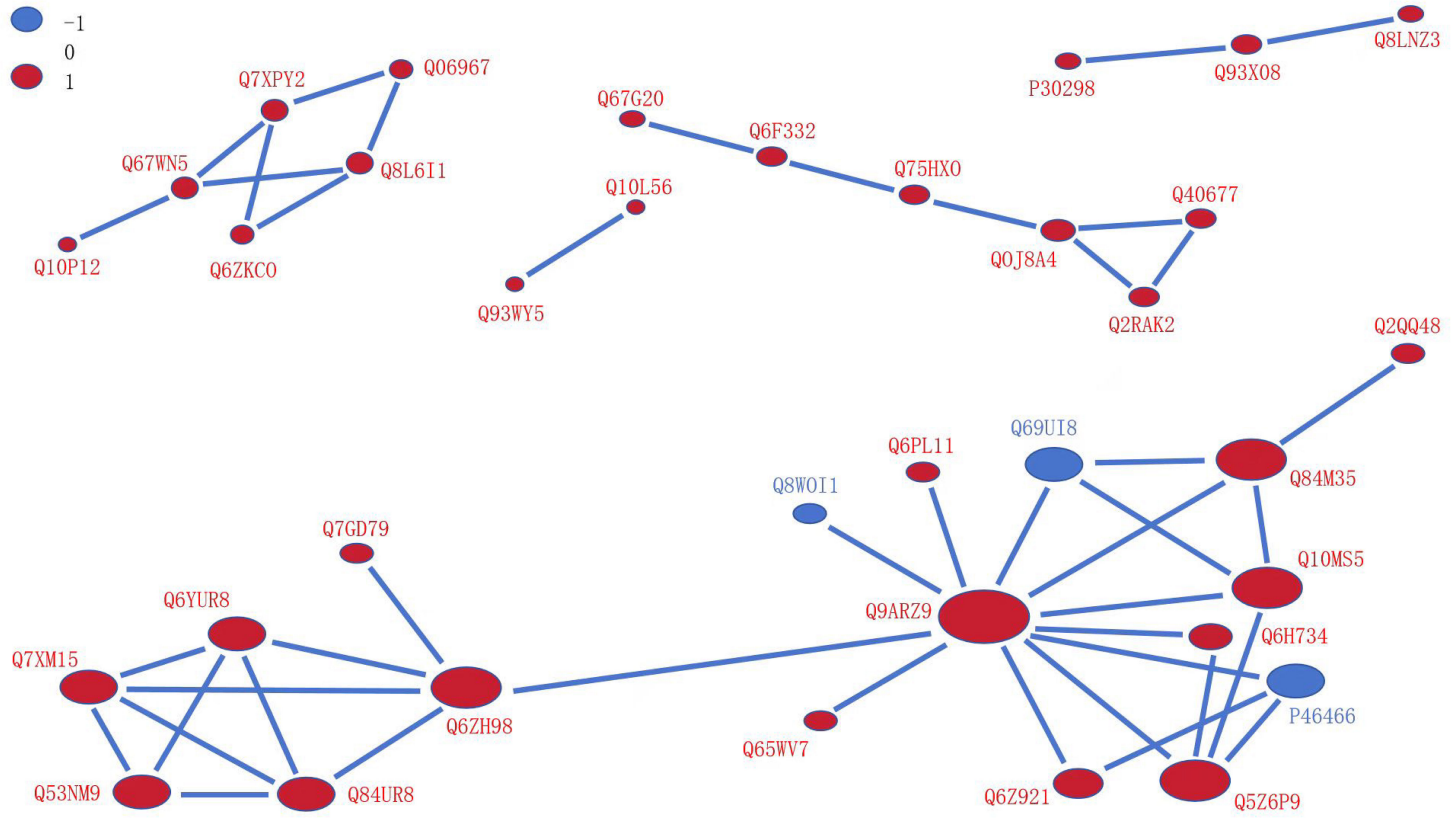
Protein–protein interaction network for DUMPs. The circles represent differentially modified proteins, and different colors represent the differentially modified proteins (blue indicates the downregulated protein, and red indicates the upregulated protein). The size of circle represents the number of differentially modified proteins and their interacting proteins; the larger the circle, the higher the number. DUMPs, differentially ubiquitin-modified proteins.

We searched for proteins differentially expressed between OX24 and CK24 using the STRING (v.10.5) database for PPIs (confidence score >0.7). This comparison group had a total of 35 nodes, and of them, only three were downregulated nodes (**Figure 6**). A regulatory network of nodes was observed for Q9AZR9 (connected with 11 proteins, including Q6ZH98, Q84M35, Q10MS5, Q5Z6P9, Q6Z921, Q6H734, Q69UI8, Q6PL11, Q8W0I1, Q65WV7, and P46466), followed by Q6ZH98 (connected with six proteins), Q6YUR8, Q84UR8, Q53NM9, and Q7XM15, each connected with four proteins. A previous study showed that many proteins are closely related to cold tolerance, such as Q9AZR9 was ubiquitin-40S ribosomal protein S27a-1, Q5Z6P9 was ubiquitin receptor RAD23d, and Q8W0I1was ubiquitin-conjugating enzyme E2 36; Q6YUR8 and Q84UR8 were respectively cold shock domain protein 1 and protein 2, which play a critical role in the transcription, translation, development, and stress responses by the RNA chaperone function (Chaikam and Dale, 2008; Morino et al., 2017). Q6ZH98 (Os02g0121300), which encodes the cyclophilin 2, in cold stress inhibited *OsCYP2* expression in Shanyou 10 and Liangyoupeijiu seedlings (Ruan et al., 2011). These results suggest that the cold tolerance of *OsGRF4* is regulated by the above proteins in a complex network.

**Figure 7:**
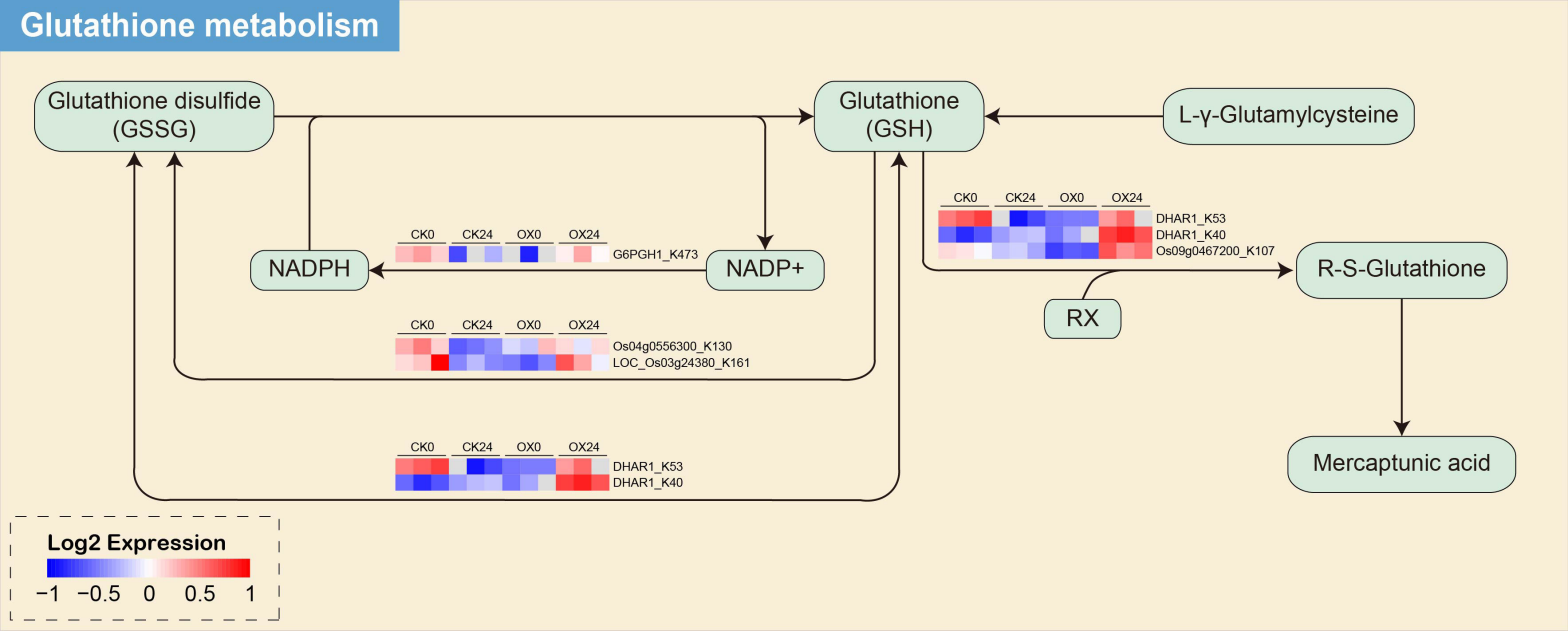
The glutathione metabolism enrichment pathway of the DUMPs in OX24 and CK24 comparison group. Red indicates the upregulated protein, and blue indicates the downregulated protein. DUMPs, differentially ubiquitin-modified proteins.

In the OX24 and CK24 comparison group, we performed the enrichment pathway in both proteome and ubiquitination modificomics (**Figure 7**, **Supplementary Figure 7**). Only two pathways of glutathione metabolism and arachidonic acid metabolism were detected in modificomics. There are five upregulated proteins in the glutathione metabolism, namely, Q93WY5, Q65XA0, Q9LI00, Q0JB49, and Q10L56; Q0JB49 and Q10L56 are upregulated in the arachidonic acid metabolism pathway. These proteins involved in glutathione transferase GST 23 (Os09g0467200), probable glutathione *S*-transferase DHAR1 (Os05g0116100), 6-phosphogluconate dehydrogenase, decarboxylating 1 (Os06g0111500), and glutathione peroxidase (Os04g0556300 and Os03g0358100). In the glutathione metabolism pathway, the five proteins were upregulated between the cyclic process of glutathione disulfide (GSSG) conversion to glutathione (GSH) and glutathione (GSH) to R-S-Glutathione process (**Figure 7**); meanwhile, glutathione peroxidase (Os04g0556300 and Os03g0358100) is also involved in the arachidonic acid metabolism pathway from the 5-HPETE to 5-HETE process and 15 (S)-HPETE to 15 (S)-HETE process (**Supplementary Figure 8**).

### 3.8 The DAPs and DUMPs co-localized with previously identified cold tolerance QTLs

**Figure 8:**
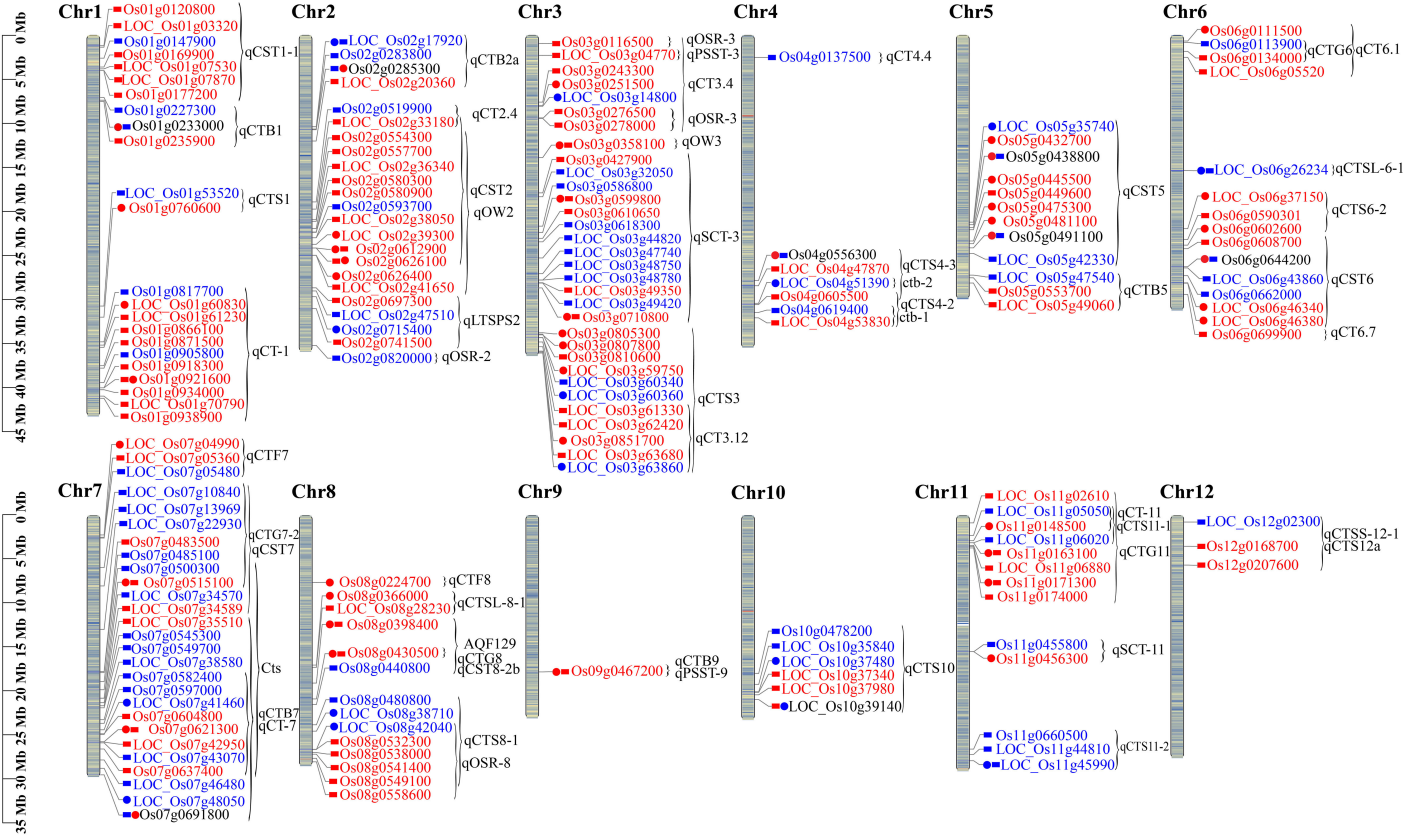
The DAPs and DUMPs co-localized with previously identified cold tolerance QTLs on rice chromosomes. The bar value on the left side is the physical location on the chromosome, the circle represents the OX24 vs. OX0 comparison group, the rectangle represents the OX24 vs. CK24 comparison group, the two shapes of the same protein indicate the common gene of the two comparison groups, red indicates upregulation, blue indicates downregulation, and the range of braces is the QTL site containing region. Os denotes a modified protein, and Loc denotes an expressed protein. DAPs, differentially abundant proteins; DUMPs, differentially ubiquitin-modified proteins.

To identify the potential function of DAPs and DUMPs, they were co-localized on chromosomes with cold or stress tolerance QTLs that have been mapped or cloned in rice. In total, 76 DAPs and 101 DUMPs were co-localized within 49 QTLs. Among them, 18 QTLs were responsible for cold tolerance at the seedling stage, seven at the reproductive stage, and two at the fertilization stage in rice, and others have been reported to be involved in abiotic and biotic tolerance in rice. Chromosome 3 had the greatest number of co-localized QTLs with nine QTLs and 32 proteins, followed by chromosome 7 with six QTLs and 27 proteins (**Figure 8**; **Supplementary Tables 11**, **12**).

The GF14f regulating cold acclimation and de-acclimation in rice (Cen et al., 2018) was co-localized with Q8H8T0 (Os03g0599800), which was upregulated in both OX24 vs. OX0 and OX24 vs. CK24 and encodes nucleotide-diphospho-sugar transferases. The result suggested that Os03g0599800 may be a candidate cold tolerance QTL. The QTLs ctb-1 (Os04g0619400) and ctb-2 (Os04g0603000) were co-localized with the glutathione peroxidase *OsGPX1* induced during cold stress (Zhao et al., 2015), and Os04g0619400 belonged to the significantly enriched KEGG pathway (**Supplementary Table 6**), and it was supposed that Os04g0619400 plays an important role in the regulatory network of rice cold stress by differential expression levels. Two proteins (Os011g0148500 and Os11g0147800) were co-localized in three QTLs (qCT-11, qCTS11-1, and qCTG11). Os01g0120800 and Os01g0124000 were both located in cold tolerance QTL qCST1-1 (Liu et al., 2013); Os02g0593700 was located in qCST2/qOW2 (Xia et al., 2010), which was explored in Dongxiang wild rice, and regulated cold tolerance at the seedling stage. Os08g0532800 and Os08g0532300 were both located in qCTS8-1and qOSR-8, which controlled the overwintering seedling rate in perennial glutinous rice 89-1 (Deng et al., 2018). We also found that some proteins were located in abiotic and biotic tolerance-related QTL regions. For instance, 14 proteins were co-localized in qCT2.4, qCST2, and qOW2. Of them, *OsGF14e* (Os02g0580300) was regulated by salinity, drought, and wounding (Chen et al., 2006); the upregulated OsPAL1(Os02g0626100) and Os02g0554300 encode phenylalanine ammonia-lyase and phosphatidylinositol 4-phosphatase and may be involved in plant stress tolerance. The proteins Os06g0676700, Os06g0677500, and Os06g0646600 were co-localized with *OsVP1* in qCST6. Transgenic plants overexpressing *OsVP1* and *OsNHX1* showed improved drought tolerance and salt tolerance (Liu et al., 2010). The upregulated 08g0430500 was co-localized with *HIR1*, which enhanced the resistance of plants to bacterial pathogen DC3000 (Zhou et al., 2010). In summary, the co-localized DAPs and DUMPs identified in this study provide important candidate genes for rice cold tolerance.

### 3.9 Conjoint analysis between the proteome and ubiquitination modificomics

A total of eight DAPs were detected in both proteome and ubiquitylome. Q6K6Q1, C7JA48, Q5W6Y8, and Q0JFI2 were found in the OX24 vs. OX0 comparison group; A0A0P0V9F2, A0A0P0WMW0, and Q7XM15 were detected in the OX24 vs. CK24 comparison group; Q6ZH84 was found in both the CK24 vs. CK0 and OX24 vs. OX0 comparison groups (**Table 2**). Clearly, six proteins demonstrated opposing changes in protein and ubiquitination levels, whereas the proteins Q6K6Q1 (phenylalanine ammonia-lyase) and C7JA48 (GEM-like protein 5) exhibited consistent upregulated changes in the OX24 vs. OX0 comparison group. This implies that, to a largely certain extent, the changing pattern of the proteome was opposite that of the ubiquitylome following cold stress treatment. This result suggested that proteome expression levels were mostly negatively but partly positively regulated by ubiquitination.

**Table 2:** The co-expression proteins between the ubiquitination modificomics and proteome in four comparison groups.

Protein accession	CK24 vs. CK0		OX24 vs. OX0		OX24 vs. CK24	
	LQ	LPUb	LQ	LPUb	LQ	LPUb
Q6K6Q1	Up		Up	Up		
Q6ZH84	Up	Down	Up	Down		
A0A0P0V9F2		Down		Down	Down	Up
A0A0P0WMW0					Down	Up
C7JA48		Up	Up	Up		
Q5W6Y8			Down	Up		
Q0JFI2			Down	Up	Down	
Q7XM15		Down			Down	Up

The protein Q6ZH84 (Os02g0593700) was upregulated in protein levels but downregulated in ubiquitination levels in both the CK24 vs. CK0 and OX24 vs. OX0 comparison groups. A study showed that the protein Os02g0593700 was upregulated after 48 and 72 h of cold treatment in *japonica* rice seedlings (Qing et al., 2022). Os02g0593700 is a homologous gene of NBR1. Brassinosteroids regulate NBR1-dependent selective autophagy in a BZR1-dependent manner in response to chilling stress in tomatoes (Chi et al., 2020). The prediction protein of Os02g0593700 was localized in the nucleus and categorized in cation binding of the molecular function. These results suggest that *OsGRF4* may positively regulate cold tolerance by regulating the protein expression of Os02g0593700 as a result of the downregulation of its ubiquitination.

### 3.10 PRM-based validation of DAPs

We confirmed the proteome results by PRM assay. A total of 12 proteins, including five upregulated proteins and seven downregulated proteins, were identified based on label-free quantitative analysis in the OX24 vs. OX0 comparison group. Meanwhile, 12 proteins were confirmed, including seven upregulated proteins and five downregulated proteins identified based on label-free quantitative analysis in the CK24 vs. OX24 comparison group. These proteins were selected for their functional significance concluded from proteome analysis, and the ratio of protein abundance varied in a wide range (**Table 3**; **Supplementary Figures 9**, **10**). **Table 3** shows that the overall trend of TMT and PRM is relatively consistent, except for two proteins (P40392 and Q6ZIF9) in the CK24 vs. OX24 comparison group. The results of this study will provide a new reference to screen key proteins for rice cold tolerance.

**Table 3:** PRM-based validation of DAPs.

Protein accession	Protein gene	OX0 relative abundance	OX24 relative abundance	OX24/OX0 (TMT/PRM)	OX24/OX0 *p*-Value	OX24/OX0(LQ)
Q7XXS4↓	THI1	1.49	0.51	0.34	1.66E−04	0.33
Q9SDJ2↓	ZIP1	1.26	0.74	0.59	3.58E−05	0.56
O64437↑	RINO1	0.68	1.32	1.93	2.33E−04	1.79
Q7XKF3↓	PORA	1.23	0.77	0.62	4.44E−04	0.55
Q851K1↓	Os03g0694000	1.16	0.84	0.73	1.11E−03	0.60
Q7X720↓	Os04g0518100	1.31	0.69	0.53	1.89E−04	0.48
Q10MB0↑	LOC_Os03g20120	0.45	1.55	3.42	3.95E−06	4.96
Q5VQG4↑	RFS	0.73	1.27	1.75	4.14E−04	3.02
Q8GTK0↓	P0710F09.134	1.21	0.79	0.66	4.28E−04	0.54
P12123↓	petB	1.08	0.92	0.85	3.76E−02	0.50
C7JA48↑	Os12g0478100	0.68	1.32	1.93	1.26E−03	1.60
Q6ZH84↑	Os02g0593700	0.59	1.41	2.39	1.64E−05	2.65
Protein accession	Protein gene	CK24 relative abundance	OX24 relative abundance	OX24/CK24 (TMT/PRM)	OX24/CK24 *p*-Value	OX24/CK24 (LQ)
Q7XCK6↓	Cht8	1.23	0.77	0.63	1.62E−03	0.61
Q5Z5T3↑	Os06g0567900	0.54	1.46	2.69	4.76E−05	2.00
Q75IQ4↓	OEP24	1.17	0.83	0.71	8.37E−08	0.54
Q0JG30↑	Os01g0946500	0.61	1.39	2.29	7.42E−07	1.61
Q8L5K0↑	Fer2	0.77	1.23	1.59	1.44E−04	1.55
P40392↓	RIC1	0.97	1.03	1.06	3.55E−01	0.64
Q5VQL0↑	P0691E06.11-2	0.91	1.09	1.19	2.16E−03	1.75
Q653V4↑	B1153E06.13-1	0.74	1.26	1.71	1.53E−03	1.85
Q0JK51↓	Os01g0695800	1.17	0.83	0.71	9.17E−05	0.62
Q0E217↑	Os02g0280500	0.99	1.01	1.02	5.67E−01	0.65
A0A0P0WMW0↓	Os05g0432700	1.12	0.88	0.79	1.71E−03	0.57
Q6ZIF9↑	CYL1	1.01	0.99	0.97	6.07E−01	3.60

DAPs, differentially abundant proteins.

↑: Upregulated ↓: Downregulated.

## 4 Discussion

### 4.1 Effects of various modifications on abiotic stress of plants

Protein PTMs regulate large-scale biological processes such as cell cycle, metabolism, and signal transduction in plants (Lehtimaki et al., 2015; Hashiguchi and Komatsu, 2016; Walsh et al., 2005). Light regulates plant growth and development through SUMOylation-mediated protein stability, SEU is a substrate of SUMO1, and the substitution of four conserved lysine residues disrupts the SUMOylation of SEU and impairs its function in photo- and thermomorphogenesis (Zhang et al., 2020a). Under chilling stress, OsSAPK6 could phosphorylate IPA1 and increase its stability. Genetic evidence showed that OsSAPK6, IPA1, and OsCBF3 were all positive regulators of rice chilling tolerance (Jia et al., 2022). At the late stage of cryogenic response, the ubiquitination chain modification of the K48 link of ICE1 protein significantly increased, leading to protein degradation and resulting in the downregulation of *CBF* gene expression (Wang et al., 2023). In this study, overexpression of *OsGRF4* could positively regulate the rice cold tolerance by ubiquitination; a total of 178 sites were quantified as upregulated and 92 sites were quantified as downregulated DUMPs in OX24 vs. OX0 in response to cold. For example, E3 SUMO-protein ligase SIZ1 (Os05g0125000) was upregulated 1.79-fold in OX24/0X0, the SUMO protein has a potential role in plant development under cold stress, and the process of SUMOylation is very similar to that of ubiquitination (Chaikam, 2009). *S*-Adenosylmethionine synthase 2 (Os051g0323600) was significantly downregulated in OX24 vs. OX0, which regulates the cold-responsive gene expression by histone H3K27ac and H3K27me3 modifications (Dasgupta et al., 2022). Similarly, 82 sites were identified as upregulated and 13 sites were identified as downregulated DUMPs in CK24 vs. OX24. Some critical pathways and protein domains were found in the proteomic and ubiquitination combined analysis, such as propanoate metabolism, cysteine and methionine metabolism, pyruvate metabolism, and glutathione metabolism. Aspartate aminotransferase (Os01g0760600), beta-glucosidase BoGH3B (Os05g0449600), and OsVP1 (Os05g0449600) were upregulated and involved in cold tolerance (Alamholo and Tarinejad, 2024; Zhang et al., 2011). Similarly, rice responds to oxidative stress by changing its own acetylation and succinylation modifications; a total of 2,593 succinylated proteins (5,502 sites) and 1,024 acetylated proteins (1,669 sites) were identified in rice leaves, of which 723 proteins were both acetylated and succinylated (Zhou et al., 2017). Also, there are translation elongation factor, GRAM domain, ubiquitin-associated domain, the cold shock protein, Zinc finger, and 14-3-3 domain enriched in the two comparison groups. *OsGRF4* may be the focus to connect these pathways and regulate the rice cold tolerance.

### 4.2 Effect of ubiquitination modification on cold tolerance in rice

Ubiquitination modification plays a key role in rice defense against stress. OST1 (OPEN STOMATA 1) interacts with both the transcription factor ICE1 and the E3 ligase HOS1 in the *CBF* pathway. OST1 interferes with the interaction between HOS1 and ICE1, thus suppressing HOS1-mediated ICE1 ubiquitination degradation under cold stress (Ding et al., 2015). OsMAPK3 phosphorylates OsbHLH002/OsICE1 and inhibits its ubiquitination to activate OsTPP1 and enhances rice chilling tolerance (Zhang et al., 2017). Both PUB25 and PUB26 poly-ubiquitinate MYB15, a transcriptional repressor of the *CBF*-dependent cold signaling pathway, leading to MYB15 degradation and thus enhanced *CBF* expression under cold stress (Wang et al., 2019a). These studies have shown that ubiquitin-conjugating enzyme E2 was downregulated and ubiquitin-40S ribosomal protein S27a-1 was upregulated in the OX24 vs. CK24 comparison group; ubiquitin carboxyl-terminal hydrolase, ubiquitin receptor RAD23d, ubiquitin receptor RAD23b isoform X2, E3 SUMO-protein ligase SIZ1, and ubiquitin-40S ribosomal protein S27a-1 were upregulated in the OX24 vs. OX0 comparison group. Eight new proteins were changed by combining proteomic and ubiquitinomic analyses. Phenylalanine ammonia-lyase (Os02g0626400), cysteine proteinase inhibitor (Os05g0432700), subtilisin-like protease SBT3.17 (Os12g0478200), GEM-like protein 5 (Os05g0432700), universal stress protein A-like protein (Os05g0355400), Cytochrome P450 (Os04g0101400), PHD finger-like domain-containing protein 5B (Os04g0663300), and protein NBR1 homolog isoform X1(Os02g0593700). These novel genes are likely involved in rice cold tolerance through ubiquitination. Verifiably, the protein accession Q6ZH84 matches the gene Os02g0593700, prediction was localized in the nucleus and categorized in cation binding of the molecular function in our results, and Os02g0593700 was upregulated in protein levels but downregulated in ubiquitination levels in both the CK24 vs. CK0 and OX24 vs. OX0 comparison groups, which suggested that OsGRF4 may positively regulate cold tolerance by upregulating the protein expression of Os02g0593700, resulting from the downregulation of its ubiquitination.

### 4.3 Photosynthetic system-related genes can adapt to cold stress by reducing the energy consumption of photosynthetic system

Under abiotic stress, rice photosynthesis-related genes changed to adapt to the hostile environment. The light signal transduction pathway PIF3 is involved in the negative regulation of plant frost resistance, and the E3 ubiquitination ligase EBF1/EBF2 is involved in low temperature-mediated regulation of PIF3 protein stability, revealing the molecular mechanism of light signal involved in the negative regulation of plant frost resistance (Jiang et al., 2017, Jiang et al., 2020). Porphyrin and chlorophyll metabolism genes were induced by daytime chilling stress but repressed by nighttime chilling stress, such as YGL8 (yellow-green leaf 8), LYL1 (light-induced yellow leaf 1), OsPORA (protochlorophyllide-oxidoreductase A), OsCAO1 (copper amine oxidase 1), and SGRL (stay-green rice like), playing key regulating roles in chlorophyll metabolism (Lu et al., 2021). The rice aspartyl-tRNA synthetase YLC3 regulates amino acid homeostasis and chloroplast development under low temperatures (Liu et al., 2022). A total of 11 different expression proteins related to photosynthetic system in rice cold tolerance, Q5VN31,1,4-alpha-glucan-branching enzyme 3, was found in both the CK24 vs. OX24 and OX24 vs. OX0 comparison groups (**Table 4**). All these proteins were downregulated in the comparison groups. Of these photosynthetic system-related proteins, nine proteins were located in the chloroplast, and only P12123 and A0A0N7KS13 were located in the cytoplasm. *OsGRF4* may be involved in photosynthesis with these genes to regulate cold tolerance in rice.

**Table 4:** The proteins involved in photosynthesis in comparison groups.

Protein accession	Protein description	Comparison group
Q5ZA98	Chlorophyll a-b binding protein	CK24 vs. OX24
Q5VN31	1,4-Alpha-glucan-branching enzyme 3	CK24 vs. OX24; OX24 vs. OX0
Q5VRI5	Cytochrome P450 93G2	OX24 vs. OX0
Q9SDJ2	Magnesium-protoporphyrin IX monomethyl ester	OX24 vs. OX0
Q7XKF3	Protochlorophyllide reductase A	OX24 vs. OX0
Q0J8R9	Photosystem II 22 kDa protein	OX24 vs. OX0
P12123	Cytochrome b6	OX24 vs. OX0
Q84PB5	Photosystem I reaction center subunit psaK	OX24 vs. OX0
Q0JFI2	Cytochrome P450 93G1	OX24 vs. OX0
A0A0N7KS13	Glutamyl-tRNA reductase	OX24 vs. OX0
Q7G602	Flavonoid 3′-monooxygenase CYP75B3	OX24 vs. OX0

### 4.4 Effect of transcription factors on cold tolerance in rice

Transcription factors, such as Zingers, NACs, and MYBs, regulate the tolerance of cold stress in rice. *OsMYB30* interacts with *OsJAZ9* to repress the expression of *β-AMYLASE*, leading to reduced levels of the osmoprotectant maltose and thereby negatively regulating cold tolerance (Lv et al., 2017). The transcription factor *OsMADS57* interacts with *OsTB1* to regulate the trade-off between plant growth and chilling tolerance in rice (Chen et al., 2018). Moreover, the cyclophilin *OsCYP20-2* also participates in the balance between chilling tolerance and cell elongation by interacting with *SLR1* and *OsFSD2* (Ge et al., 2020). In this study, some vital transcription factors were explored in DUMPs, such as cold shock domain (Q6YUR8) and NAC(Q8RUI4) in the OX24/OX0 group, and Myb protein 1 (Q65WV7), FAM63A(Q5Z800), ABC(Q0JQA5), remorin (Q7XMK5), and Zinger(Q688R3) in the OX24/CK24 group. These proteins may be involved in the regulation of cold tolerance in rice. Some transcription factors have pleiotropic genes involved in rice cold tolerance and other stress resistance. The transcription factor *Ghd8* controls grain number, plant height, and heading date in rice. Transgenic analyses revealed that enhanced expression levels of *Ghd8* delayed heading date and enhanced cold tolerance in rice (Wang et al., 2019b). *OsWRKY53* functions as a transcriptional repressor, playing an important role in plant growth development and response to various stresses. Knockout of *OsWRKY53* thickens sclerenchyma cell walls, confers bacterial blight resistance and sheath blight resistance, and regulates rice cold tolerance at the booting stage by fine-tuning anther gibberellin levels (Xie et al., 2021; Yuan et al., 2020; Gao et al., 2020; Yang et al., 2022; Tang et al., 2022). Recently, Yu et al. identified *OsWRKY53* as a key regulator of salt tolerance in rice through a genome-wide association study, and the CRISPR knockout line Cr-oswrky53 had a higher fresh weight rate and survival rate after salt stress treatment, while the overexpression line had the opposite effect (Yu et al., 2023). *OsGRF4* controls grain development by regulating brassinosteroid-induced genes, and disruption of the *miR396* target site in *OsGRF4* enhances grain size and yield in rice, which also balances the inhibitory activities of DELLA to promote and integrate nitrogen assimilation, carbon fixation, and growth (Hu et al., 2015; Che et al., 2015; Sun et al., 2016; Li et al., 2018; Chen et al., 2019). Knockout of MIR396ef (MIR396e and MIR396f) enhanced both grain size and panicle branching, resulting in increased grain yield. Importantly, under nitrogen-deficient conditions, mir396ef mutants showed an even higher relative increase in grain yield as well as elevated above-ground biomass (Zhang et al., 2020b). Therefore, we speculate that *OsGRF4* may have other stress resistance functions in addition to cold tolerance.

## 5 Conclusion

In this study, a ubiquitin-associated protein LOC_Os02g38050 was upregulated and found in the OX24 vs. OX0 protein comparison group. In the ubiquitination modification group, OX24 vs. OX0 was downregulated, and CK24 vs. CK0 was also downregulated. LOC_Os02g38050 is a homolog of the NBR1 cold tolerance gene. Therefore, we speculate that LOC_Os02g38050 should affect the expression of protein through ubiquitination modification, thus affecting cold tolerance.

## Data Availability

The datasets presented in this study can be found in online repositories. The names of the repository/repositories and accession number(s) can be found in the article/**Supplementary Material.**

## References

[B1] Alamholo M. Tarinejad A. (2024). Molecular mechanism of salinity tolerance in barley (*Hordeum vulgare* L.) via meta-analysis of transcriptome data. J. Agric. Sci. Technol. 26, 111–126.

[B2] Cen W. Liu J. Lu S. Ye Y. Yu J. Fu X. (2018). Comparative proteomic analysis of QTL CTS-12 derived from wild rice (*Oryza rufipogon* Griff.), in the regulation of cold acclimation and de-acclimation of rice. Nature 560, 595–600. doi: 10.1186/s12870-018-1381-7 30097068 PMC6086036

[B3] Chaikam V. V. R. (2009). Characterization of cold shock domain proteins and SUMOylation system from *Oryza sativa.* Graduate Theses (Morgantown: West Virginia University).

[B4] Chaikam V. Dale K. (2008). Functional characterization of two cold shock domain proteins from *Oryza sativa* . Plant Cell Environ. 31, 995–1006. doi: 10.1111/j.1365-3040.2008.01811.x 18397370

[B5] Che R. Tong H. Shi B. Liu Y. Fang S. Chu C. (2015). Control of grain size and rice yield by GL2-mediated brassinosteroid responses. Nat. Plants 2, 15195. doi: 10.1038/nplants.2015.195 27250747

[B6] Chen Y. Chen Z. Kang J. Kang D. Gu H. Qin G.. (2013). AtMYB14 regulates cold tolerance in arabidopsis. Plant Mol. Biol. Rep. 31, 87–97. doi: 10.1007/s11105-012-0481-z PMC3881570 24415840

[B7] Chen R. Z. Deng Y. W. Ding Y. L. Guo J. X. Qiu J. Wang B.. (2022). Rice functional genomics: decades’ efforts and roads ahead. Sci. China (Life Sci.) 65, 33–92. doi: 10.1007/s11427-021-2024-0 34881420

[B8] Chen X. L. Jiang L. R. Zheng J. S. Chen F. Y. Wang T. S. Wang M. L.. (2019). A missense mutation in Large Grain Size 1 increase grain size and enhances cold tolerance in rice. J. Exp. Bot. 70, 3851–3866. doi: 10.1093/jxb/erz192 31020332 PMC6685659

[B9] Chen F. Li Q. Sun L. He Z. (2006). The rice 14-3-3 gene family and its involvement in responses to biotic and abiotic stress. DNA Res. 13, 53–63. doi: 10.1093/dnares/dsl001 16766513

[B10] Chen L. Zhao Y. Xu S. Zang Z. Xu Y. Zhang J.. (2018). OsMADS57 interacts with OsTB1 to regulate the trade-off between plant growth and chilling tolerance in rice. New Phytol. 218, 219–231. doi: 10.1111/nph.2018.218.issue-1 29364524 PMC5873253

[B11] Chi C. Li X. Fang P. Xia X. Shi K. Zhou Y.. (2020). Brassinosteroids act as a positive regulator of NBR1-dependent selective autophagy in response to chilling stress in tomato. J. Exp. Bot. 71, 1092–1106. doi: 10.1093/jxb/erz466 31639824

[B12] Chinnusamy V. Zhu J. Zhu J. (2007). Cold stress regulation of gene expression in plants. Trends Plant Sci. 12, 444–451. doi: 10.1016/j.tplants.2007.07.002 17855156

[B13] Corrales A. R. Carrillo L. Lasierra P. Nebauer S. G. Dominguez-Figueroa J. Renau-Morata B.. (2017). Multifaceted Role of Cycling DOF Factor (CDF) in the regulation of flowering time and abiotic stress responses in Arabidopsis. Plant Cell Environ. 40, 748–764. doi: 10.1111/pce.12894 28044345

[B14] Cui L. Min H. Byun M. Y. Oh H. G. Kim W. T. (2018). OsDIRP1, a putative RING E3 ligase, plays an opposite role in drought and cold stress responses as a negative and positive factor, respectively, in rice *(Oryza sativa* L.). Front. Plant Sci. 9. doi: 10.3389/fpls.2018.01797 PMC6290360 30568669

[B15] Dasgupta P. Prasad P. Bag S. K. Chaudhuri S. (2022). Dynamicity of histone H3K27ac and H3K27me3 modifications regulate the cold-responsive gene expression in *Oryza sativa* L. ssp. indica. Genomics 114, 110433. doi: 10.1016/j.ygeno.2022.110433 35863676

[B16] Deng X. Gan L. Liu Y. Luo A. Jin L. Chen J.. (2018). Locating QTLs controlling overwintering seedling rate in perennial glutinous rice 89-1 (*Oryza sativa* L.). Genes Genom. 40, 1351–1361. doi: 10.1007/s13258-018-0731-z 30171448

[B17] Ding Y. Li H. Zhang X. Xie Q. Gong Z. Yang S. (2015). OST1 kinase modulates freezing tolerance by enhancing ICE1 stability in Arabidopsis. Dev. Cell 32, 278–289. doi: 10.1016/j.devcel.2014.12.023 25669882

[B18] Gao Y. Xue C. Liu J. He Y. Mei Q. Wei S.. (2020). Sheath blight resistance in rice is negatively regulated by WRKY53 via SWEET2a activation. Biochem. Bioph. Res. Co. 585, 117–123. doi: 10.1016/j.bbrc.2021.11.042 34801931

[B19] Ge Q. Zhang Y. Xu Y. Bai M. Luo W. Wang B.. (2020). Cyclophilin OsCYP20-2 with a novel variant integrates defense and cell elongation for chilling response in rice. New Phytol. 225, 2453–2467. doi: 10.1111/nph.v225.6 31736073 PMC7064896

[B20] Hashiguchi A. Komatsu S. (2016). Impact of post-translational modifications of crop proteins under abiotic stress. Proteomes 4, 42. doi: 10.3390/proteomes4040042 28248251 PMC5260974

[B21] Hu J. Wang Y. Fang Y. Li J. Qian Q. (2015). A rare allele of GS2 enhances grain size and grain yield in rice. Mol. Plant 8, 1455–1465. doi: 10.1016/j.molp.2015.07.002 26187814

[B22] Ito Y. Katsura K. Maruyama K. Taji T. Kobayashi M. Motoaki S.. (2006). Functional analysis of rice DREB1/CBF-type transcription factors involved in cold-responsive gene expression in transgenic rice. Plant Cell Physiol. 47, 141–153. doi: 10.1093/pcp/pci230 16284406

[B23] Jia M. Meng X. Song X. Zhang D. Kou L. Zhang J.. (2022). Chilling-induced phosphorylation of IPA1 by OsSAPK6 activates chilling tolerance responses in rice. Cell Discovery 8, 71. doi: 10.1038/s41421-022-00413-2 35882853 PMC9325753

[B24] Jiang B. Shi Y. Peng Y. Jia Y. Yan Y. Dong X.. (2020). Cold-induced CBF-PIF3 interaction enhances freezing tolerance by stabilizing the phyBThermosensor in arabidopsis. Mol. Plant 13, 894–906. doi: 10.1016/j.molp.2020.04.006 32311530

[B25] Jiang B. Shi Y. Zhang X. Xin X. Qi L. Guo H.. (2017). PIF3 is a negative regulator of the *CBF* pathway and freezing tolerance in Arabidopsis. Proc. Natl. Acad. Sci. 114, 6695–6702. doi: 10.1073/pnas.1706226114 PMC5559041 28739888

[B26] Kosová K. Vítámvás P. Prášil I. T. Klíma M. Renaut J. (2021). Plant proteoforms under environmental stress: Functional proteins arising from a single gene. Front. Plant Sci. 12, 793113. doi: 10.3389/fpls.2021.793113 34970290 PMC8712444

[B27] Lee J. Lee W. Kwon S. W. (2015). A quantitative shotgun proteomics analysis of germinated rice embryos and coleoptiles under low-temperature conditions. Proteome Sci. 13, 27. doi: 10.1186/s12953-015-0082-5 26587012 PMC4652350

[B28] Lehtimaki N. Koskela M. M. Mulo P. (2015). Post-translational modifications of chloroplast proteins: an emerging field. Plant Physiol. 168, 768–775. doi: 10.1104/pp.15.00117 25911530 PMC4741338

[B29] Li S. Tian Y. Wu K. Ye Y. Yu J. Fu X. (2018). Modulating plant growth–metabolism co-ordination for sustainable agriculture. Nature 560, 595–600. doi: 10.1038/s41586-018-0415-5 30111841 PMC6155485

[B30] Liu H. Gong X. Deng H. Tan J. Sun Y. Wang F.. (2022). The rice aspartyl-tRNA synthetase YLC3 regulates amino acid homeostasis and chloroplast development under low temperature. Front. Plant Sci. 13, 847364. doi: 10.3389/fpls.2022.847364 36340382 PMC9635353

[B31] Liu F. Xu W. Song Q. Tan L. Liu J. Zhu Z.. (2013). Microarray-assisted fine-mapping of quantitative trait loci for cold tolerance in rice. Mol. Plant 6, 757–767. doi: 10.1093/mp/sss161 23267004

[B32] Liu S. Zheng L. Xue Y. Zhang Q. Wang L. Shou H. (2010). Overexpression of osVP1 and osNHX1 increases tolerance to drought and salinity in rice. J. Plant Biol. 53, 444–452. doi: 10.1007/s12374-010-9135-6

[B33] Lu X. Song S. Xiao Y. Fan F. Yan Z. Jia G.. (2021). Circadian clock-coordinated response to chilling stress in rice. Environ. Exp. Bot. 185, 104398. doi: 10.1016/j.envexpbot.2021.104398

[B34] Lu G. Wu F. Wu W. Wang H. J. Zheng X. Zhang Y.. (2014). Rice LTG1 is involved in adaptive growth and fitness under low ambient temperature. Plant J. 78, 468–480. doi: 10.1111/tpj.2014.78.issue-3 24635058

[B35] Lv Y. Yang M. Hu D. Yang Z. Ma S. Li X.. (2017). The *OsMYB30* transcription factor suppresses cold tolerance by interacting with a JAZ protein and suppressing β-amylase expression. Plant Physiol. 173, 1475–1149. doi: 10.1104/pp.16.01725 28062835 PMC5291022

[B36] Ma Y. Dai X. Xu Y. Luo W. Zheng X. Zeng D.. (2015). COLD1 confers chilling tolerance in rice. Cell 160, 1209–1221. doi: 10.1016/j.cell.2015.01.046 25728666

[B37] Mao D. Xin Y. Tan Y. Hu X. Bai J. Liu Z.. (2019). Natural variation in the HAN1 gene confers chilling tolerance in rice and allowed adaptation to a temperate climate. Proc. Natl. Acad. Sci. 116, 3494–3501. doi: 10.1073/pnas.1819769116 30808744 PMC6397538

[B38] Morino K. Kimizu M. Fujiwara M. (2017). Disulfide proteomics of rice cultured cells in response to OsRacl and probenazolerelated immune signaling pathway in rice. Proteome Sci. 15, 6. doi: 10.1186/s12953-017-0115-3 28413359 PMC5390479

[B39] Neilson K. A. Mariani M. Haynes P. A. (2011). Quantitative proteomic analysis of cold-responsive proteins in rice. Proteomics 11, 1696–1706. doi: 10.1002/pmic.201000727 21433000

[B40] Qing D. Deng G. Pan Y. Gao L. Liang H. Zhou W.. (2022). ITRAQ-based quantitative proteomic analysis of japonica rice seedling during cold stress. Breed. Sci. 72, 150–168. doi: 10.1270/jsbbs.21081 36275934 PMC9522529

[B41] Ruan S. L. Ma H. S. Wang S. H. Fu Y. P. Xin Y. Liu W. Z.. (2011). Proteomic identification of OsCYP2, a rice cyclophilin that confers salt tolerance in rice (*Oryza sativa* L.) seedlings when overexpressed. BMC Plant Biol. 11, 34. doi: 10.1186/1471-2229-11-34 21324151 PMC3050798

[B42] Saito K. Hayano-Saito Y. Kuroki M. Sato Y. (2010). Map-based cloning of the rice cold tolerance gene CTB1. Plant Sci. 179, 97–102. doi: 10.1016/j.plantsci.2010.04.004

[B43] Sun P. Zhang W. Wang Y. Deng H. (2016). OsGRF4 controls grain shape, panicle length and seed shattering in rice. J. Integr. Plant Biol. 58, 836–847. doi: 10.1111/jipb.v58.10 26936408 PMC5089622

[B44] Tang J. Tian X. Mei E. He M. Gao J. Yu J.. (2022). *WRKY53* negatively regulates rice cold tolerance at the booting stage by fine-tuning anther gibberellin levels. Plant Cell 34, 4495–4515. doi: 10.1093/plcell/koac253 35972376 PMC9614489

[B45] Vu L. D. Gevaert K. De Smet I. (2018). Protein language: Post-translational modifications talking to each other. Trends Plant Sci. 23, 1068–1080. doi: 10.1016/j.tplants.2018.09.004 30279071

[B46] Walsh C. T. Garneau-Tsodikova S. Gatto G. J. (2005). Protein posttranslational modifications: the chemistry of proteome diversifications. Angew. Chem. 44, 7342–7372. doi: 10.1002/anie.200501023 16267872

[B47] Wang X. Ding Y. Li Z. Shi Y. Wang J. Hua J.. (2019a). PUB25 and PUB26 promote plant freezing tolerance by degrading the cold signaling negative regulator. Dev. Cell 51, 222–235. doi: 10.1016/j.devcel.2019.08.008 31543444

[B48] Wang Z. Sun J. Zu X. Gong J. Deng H. Hang R.. (2022). Pseudouridylation of chloroplast rRNA contributes to low temperature acclimation in rice. New Phytol. 236, 1708–1720. doi: 10.1111/nph.v236.5 36093745

[B49] Wang J. Wang J. Wang X. Li R. Chen B. (2018). Proteomic response of hybrid wild rice to cold stress at the seedling stage. PloS One 13, e0198675. doi: 10.1371/journal.pone.0198675 29879216 PMC5991693

[B50] Wang P. Xiong Y. Gong R. Yang Y. Fan K. Yu S. (2019b). A key variant in the cis-regulatory element of flowering gene *Ghd8* associated with cold tolerance in rice. Sci. Rep. 9, 1–14. doi: 10.1038/s41598-019-45794-9 PMC6610134 31270366

[B51] Wang X. Zhang X. Y. Song C. P. Gong Z. Z. Yang S. H. Ding Y. L. (2023). PUB25 and PUB26 dynamically modulate ICE1 stability via differential ubiquitination during cold stress in Arabidopsis. Plant Cell 35, 3585–3603. doi: 10.1093/plcell/koad159 37279565 PMC10473228

[B52] Xia R. Xiao N. Hong Y. Zhang C. Su Y. Zhang X.. (2010). QTLs mapping for cold tolerance at seedling stage in Dongxiang wild rice (*Oryza rufipogon* Griff.). Sci. Agric.Sin. 43, 443–451. doi: 10.3864/j.issn.0578-1752.2010.03.001

[B53] Xie X. Kang H. Liu W. Wang G. (2015). Comprehensive profiling of the rice ubiquitome reveals the significance of lysine ubiquitination in young leaves. J. Proteome Res. 14, 2017–2025. doi: 10.1021/pr5009724 25751157

[B54] Xie W. Ke Y. Cao J. Wang S. Yuan M. (2021). Knock out of transcription factor *WRKY53* thickens sclerenchyma cell walls, confers bacterial blight resistance. Plant Physiol. 187, 1746–1761. doi: 10.1093/plphys/kiab400 34618083 PMC8566205

[B55] Yang S. Fu Y. W. Zhang Y. Yuan D. P. Li S. Kumar V.. (2022). Rhizoctonia solani transcriptional activator interacts with rice WRKY53 and grassy tiller 1 to activate SWEET transporters for nutrition. J. Adv. Res. 50, 1–12. doi: 10.1016/j.jare.2022.10.001 PMC10403663 36252923

[B56] Yu J. Zhu C. Xuan W. An H. Tian Y. Wang B.. (2023). Genome-wid association studies identify *OsWRKY53* as a key regulator of salt tolerancece in rice. Nat. Commun. 14, 1–13. doi: 10.1038/s41467-023-39167-0 PMC10272163 37321989

[B57] Yuan D. Xu X. Hong W. Wang S. Jia X. Liu Y.. (2020). Transcriptome analysis of rice leaves in response to Rhizoctonia solani infection and reveals a novel regulatory mechanism. Plant Biotechnol. Rep. 14, 559–573. doi: 10.1007/s11816-020-00630-9

[B58] Zhang X. Hua J. Liu S. Jin B. Lin R. (2020a). SIZ1-mediated SUMOylation of ROS1 enhances its stability and positively regulates active DNA demethylation in arabidopsis. Plant Commun. 1, 1–12. doi: 10.1016/j.xplc.2020.100080 32927102

[B59] Zhang J. Zhou Z. Bai J. Tao X. Wang L. Zhang H.. (2020b). Disruption of *MIR396e* and *MIR396f* improves rice yield undernitrogen-deficient conditions. Natl. Sci. Rev. 7, 102–112. doi: 10.1093/nsr/nwz142 34692021 PMC8288854

[B60] Zhang Z. Li J. Li F. Liu H. Yang W. Chong K.. (2017). OsMAPK3 phosphorylates OsbHLH002/OsICE1 and inhibits its ubiquitination to activate OsTPP1 and enhances rice chilling tolerance. Dev. Cell 43, 731–743. doi: 10.1016/j.devcel.2017.11.016 29257952

[B61] Zhang J. Li J. Wang X. Chen J. (2011). OVP1, a Vacuolar ~+-translocating inorganic pyrophosphatase (V-PPase), overexpression improved rice cold tolerance. Plant Physiol. Bioch. 49, 33–38. doi: 10.1016/j.plaphy.2010.09.014 20974539

[B62] Zhang W. Sun P. He Q. Shu F. Deng H. (2013). Fine mapping of GS2, a dominant big-grain gene in rice. Crop J. 1, 160–165. doi: 10.1016/j.cj.2013.10.003

[B63] Zhang W. Sun P. He Q. Shu F. Deng H. (2018). Transcriptome analysis of near-isogenic line provides novel insights into genes associated with panicle traits regulation in rice. PloS One 13, e0199077. doi: 10.1371/journal.pone.0199077 29924832 PMC6010284

[B64] Zhao C. Zhang Z. Xie S. Si T. Li Y. Zhu J. (2016). Mutational evidence for the critical role of CBF transcription factors in cold acclimation in Arabidopsis. Plant Physiol. 171, 2744–2759. doi: 10.1104/pp.16.00533 27252305 PMC4972280

[B65] Zhao J. Zhang S. Yang T. Zeng Z. Huang Z. Zeng Z.. (2015). Global transcriptional profiling of a cold-tolerant rice variety under moderate cold stress reveals different cold stress. Physiol. Plantarum. 154, 381–394. doi: 10.1111/ppl.2015.154.issue-3 25263631

[B66] Zhou L. Cheung M. Li M. Fu Y. Sun Z. Sun S.. (2010). Rice Hypersensitive Induced Reaction Protein 1 (OsHIR1) associates with plasma membrane and triggers hypersensitive cell death. BMC Plant Biol. 10, 290. doi: 10.1186/1471-2229-10-290 21192820 PMC3022912

[B67] Zhou H. Finkemeier I. Guan W. Tossounian M. A. Wei B. Young D.. (2017). Oxidative stress-triggered interactions between the succinyl- and acetyl-proteomes of rice leaves. Plant Cell Environ. 41, 1139–1153. doi: 10.1111/pce.13100 29126343

