# Supplementary material for: Proteomes and ubiquitylomes reveal the regulation mechanism of cold tolerance mediated by OsGRF4 in rice
Source: Front Plant Sci. 2025 Mar 21;16:1531399. doi: 10.3389/fpls.2025.1531399 (PMC11968423; doi:10.3389/fpls.2025.1531399)
Supplement: Supplementary file 1 [file DataSheet1.pdf]

Supplemental Figures

Fig. S1 Two-dimensional scatter plot for principal component analysis of protein quantification between repeated samples.

Fig. S2 Box-plot of protein quantitative RSD distribution between repeated samples.

Fig. S3 Functional enrichment analysis of DAPs with up-regulated (A) and down-regulated (B).

Fig. S4 Box-plot of the distribution of qutantitative RSDmodified by ubiquitination between repeated samples.

Fig. S5 MS/MS spectrum database search analysis summary.

Fig. S6 The COG functional classification of DUMPs in OX24/OX0 and OX24/CK24 comparison groups. Captial letters on the x-axis represent the KOG categories listed to the right of the hist ogram, and the y-axis represents the number of DUMPs.

Fig. S7 KEGG pathway functional enrichment cluster of DUMPs in four comparison groups.

Fig. S8 The arachidonic acid metabolism enrichment pathway of the DUMPs in OX24 and CK24 comparison group. The red is the up-regulated protein, green is the down-regulated protein.

Fig. S9 Parallel reaction monitoring (PRM) verified the ion peak area of 12

proteins peptide in OX24 and CK24 comparison group

Fig. S10 Parallel reaction monitoring (PRM) verified the ion peak area of 12 proteins peptide in OX24 vs OX0 comparison group

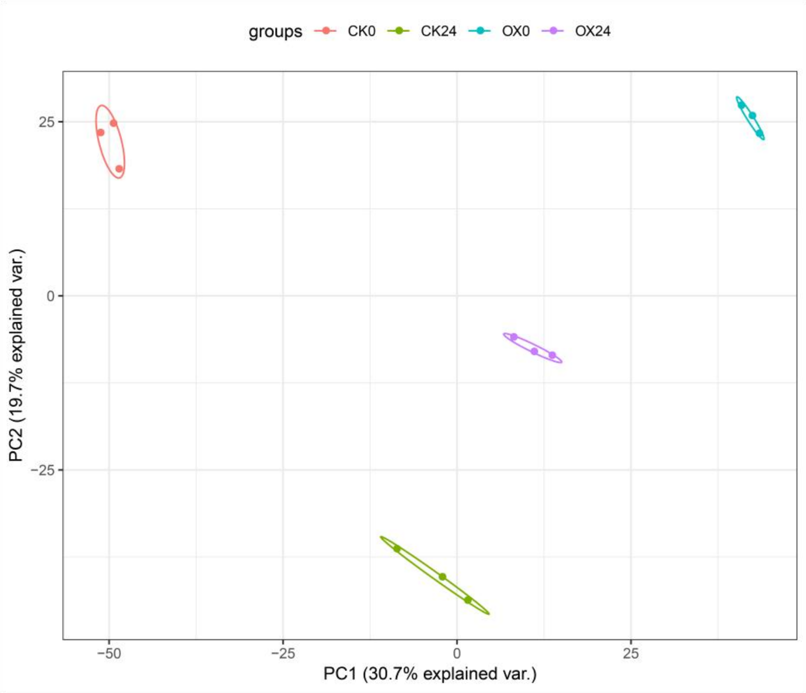

Fig. S1

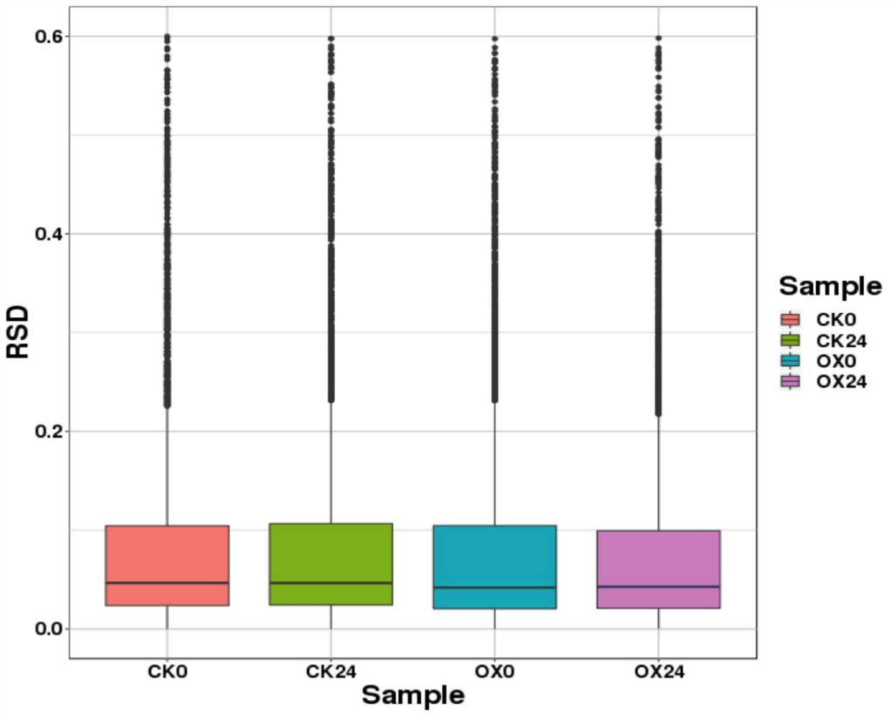

Fig. S2

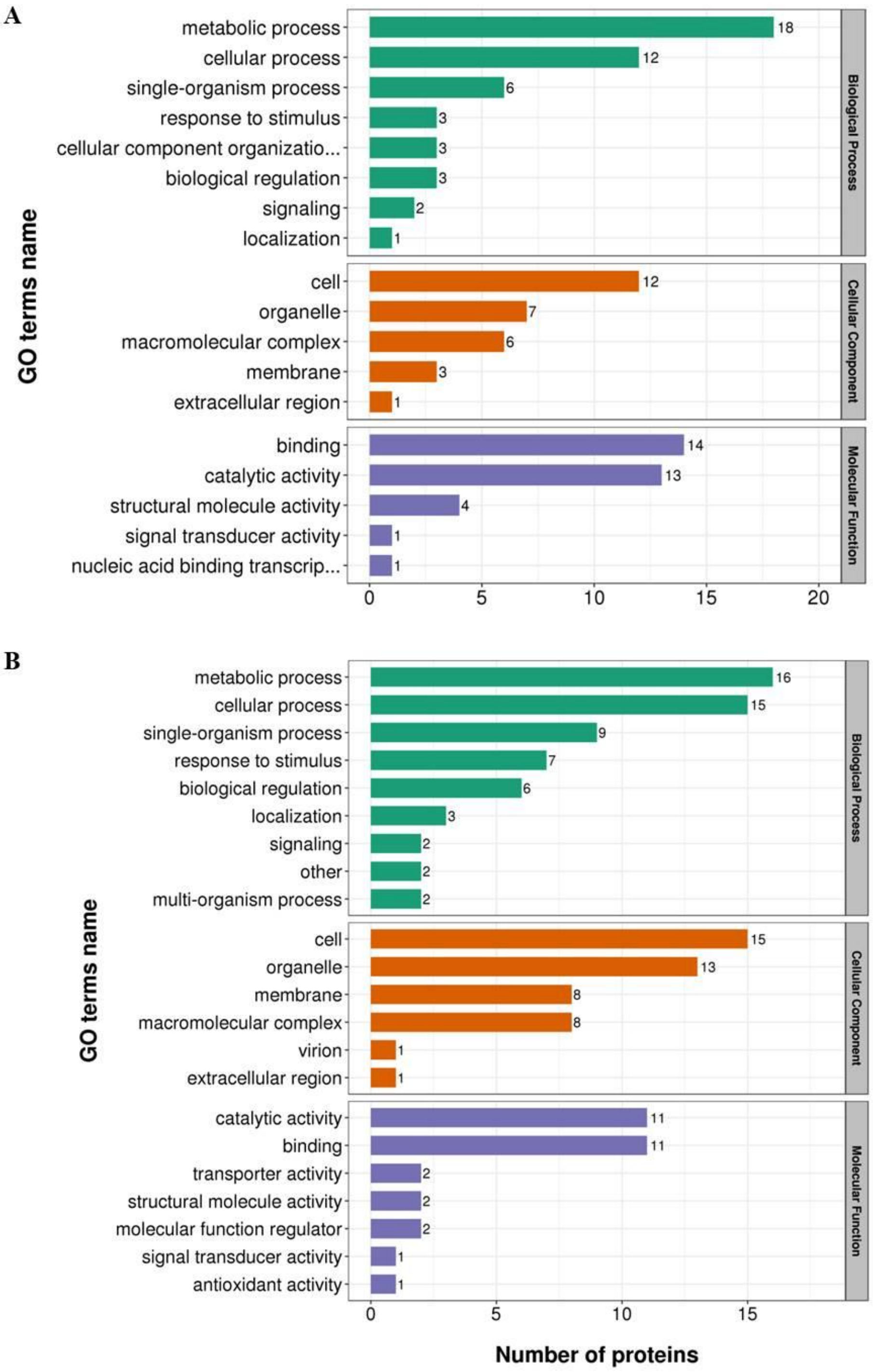
A
metabolic process
cellular process
single-organism process
response to stimulus
cellular component organizatio...
biological regulation
signaling
localization
cell
organelle
macromolecular complex
membrane
extracellular region
binding
catalytic activity
structural molecule activity
signal transducer activity
nucleic acid binding transcrip...
GO terms name
Biological Process
Cellular Component
Molecular Function
18
12
6
3
3
3
2
1
12
7
6
3
1
14
13
4
1
1
0
5
10
15
20
B
metabolic process
cellular process
single-organism process
response to stimulus
biological regulation
localization
signaling
other
multi-organism process
cell
organelle
membrane
macromolecular complex
virion
extracellular region
catalytic activity
binding
transporter activity
structural molecule activity
molecular function regulator
signal transducer activity
antioxidant activity
GO terms name
Biological Process
Cellular Component
Molecular Function
16
15
9
7
6
3
2
2
2
15
13
8
8
1
1
11
11
2
2
2
1
1
0
5
10
15
Number of proteins

Fig. S3

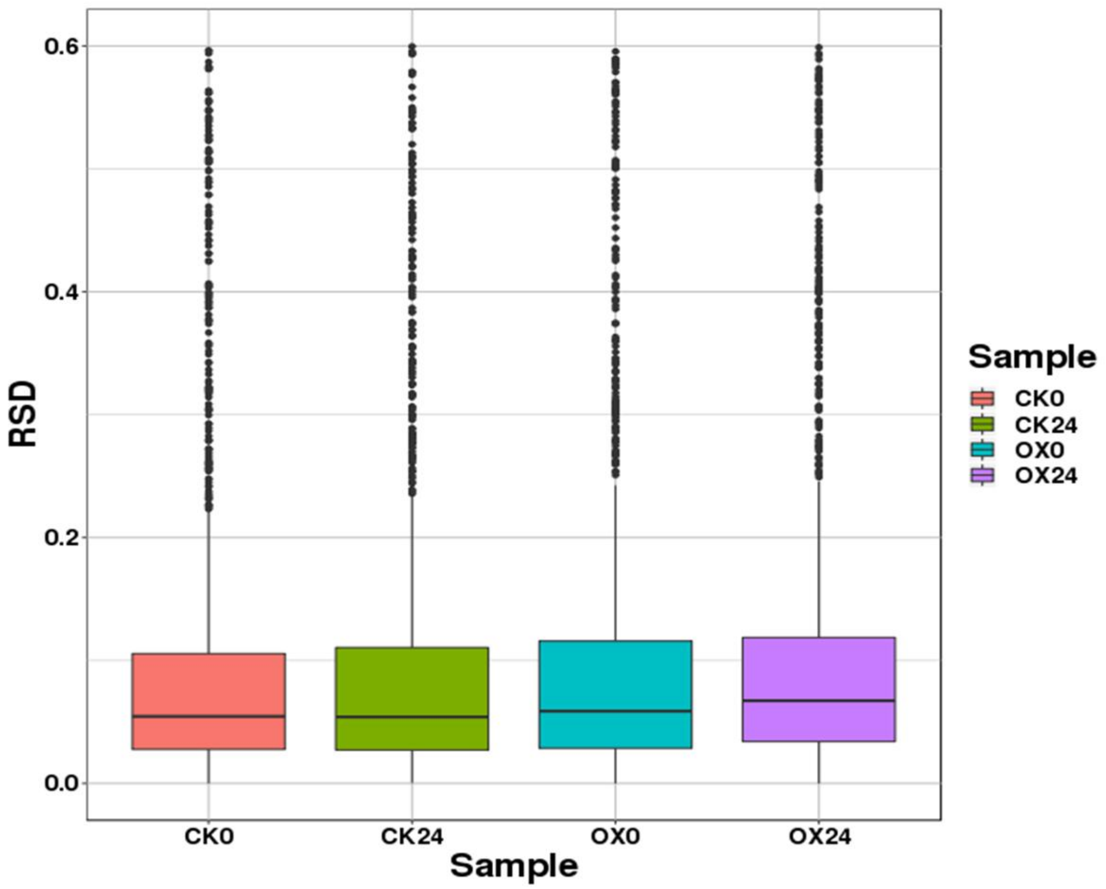
0.6
0.4
0.2
0.0
RSD
CK0
CK24
OX0
OX24
Sample
Sample
CK0
CK24
OX0
OX24

Fig. S4

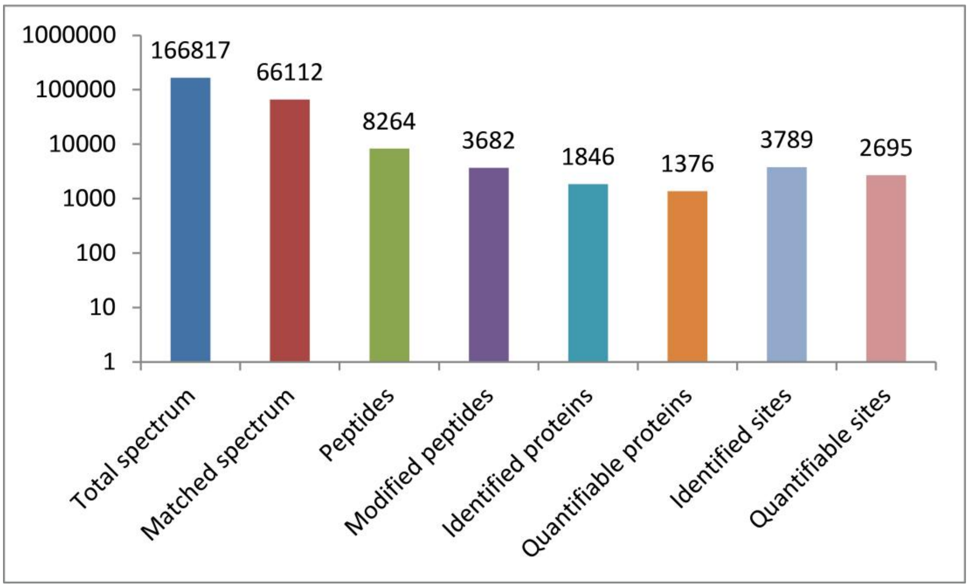
1000000
100000
10000
1000
100
10
1
166817
66112
8264
3682
1846
1376
3789
2695
Total spectrum
Matched spectrum
Peptides
Modified peptides
Identified proteins
Quantifiable proteins
Identified sites
Quantifiable sites

Fig. S5

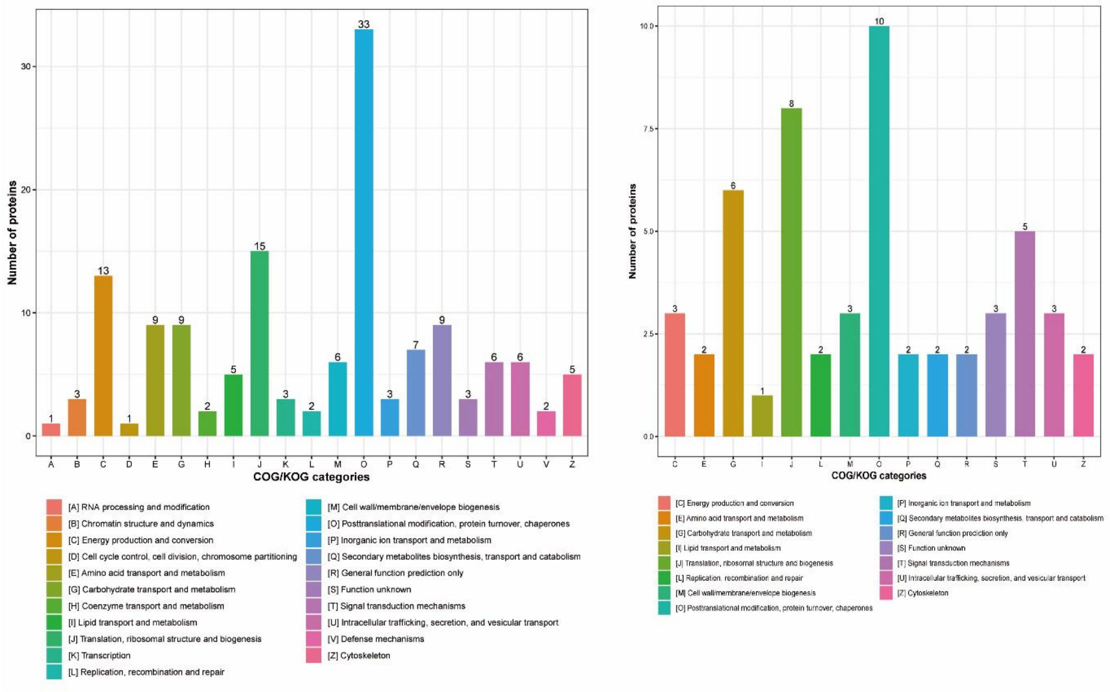
Number of proteins
COG/KOG categories
[A] RNA processing and modification
[B] Chromatin structure and dynamics
[C] Energy production and conversion
[D] Cell cycle control, cell division, chromosome partitioning
[E] Amino acid transport and metabolism
[G] Carbohydrate transport and metabolism
[H] Coenzyme transport and metabolism
[I] Lipid transport and metabolism
[J] Translation, ribosomal structure and biogenesis
[K] Transcription
[L] Replication, recombination and repair
[M] Cell wall/membrane/envelope biogenesis
[O] Posttranslational modification, protein turnover, chaperones
[P] Inorganic ion transport and metabolism
[Q] Secondary metabolites biosynthesis, transport and catabolism
[R] General function prediction only
[S] Function unknown
[T] Signal transduction mechanisms
[U] Intracellular trafficking, secretion, and vesicular transport
[V] Defense mechanisms
[Z] Cytoskeleton
Number of proteins
COG/KOG categories
[C] Energy production and conversion
[E] Amino acid transport and metabolism
[G] Carbohydrate transport and metabolism
[I] Lipid transport and metabolism
[J] Translation, ribosomal structure and biogenesis
[L] Replication, recombination and repair
[M] Cell wall/membrane/envelope biogenesis
[O] Posttranslational modification, protein turnover, chaperones
[P] Inorganic ion transport and metabolism
[Q] Secondary metabolites biosynthesis, transport and catabolism
[R] General function prediction only
[S] Function unknown
[T] Signal transduction mechanisms
[U] Intracellular trafficking, secretion, and vesicular transport
[Z] Cytoskeleton

Fig. S6

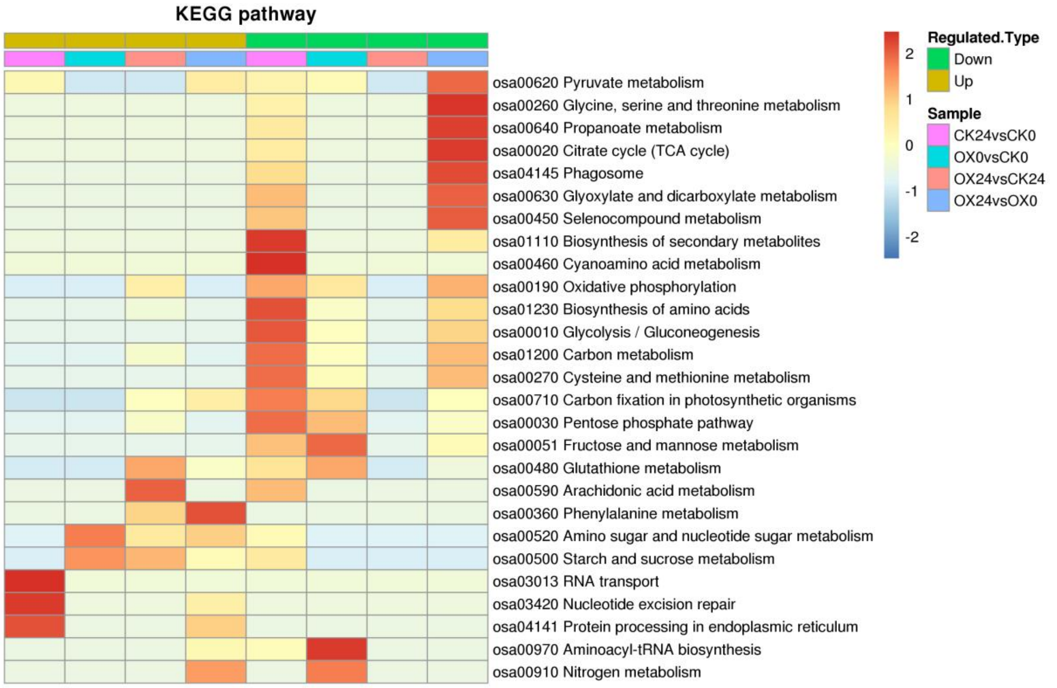
KEGG pathway
osa00620 Pyruvate metabolism
osa00260 Glycine, serine and threonine metabolism
osa00640 Propanoate metabolism
osa00020 Citrate cycle (TCA cycle)
osa04145 Phagosome
osa00630 Glyoxylate and dicarboxylate metabolism
osa00450 Selenocompound metabolism
osa01110 Biosynthesis of secondary metabolites
osa00460 Cyanoamino acid metabolism
osa00190 Oxidative phosphorylation
osa01230 Biosynthesis of amino acids
osa00010 Glycolysis / Gluconeogenesis
osa01200 Carbon metabolism
osa00270 Cysteine and methionine metabolism
osa00710 Carbon fixation in photosynthetic organisms
osa00030 Pentose phosphate pathway
osa00051 Fructose and mannose metabolism
osa00480 Glutathione metabolism
osa00590 Arachidonic acid metabolism
osa00360 Phenylalanine metabolism
osa00520 Amino sugar and nucleotide sugar metabolism
osa00500 Starch and sucrose metabolism
osa03013 RNA transport
osa03420 Nucleotide excision repair
osa04141 Protein processing in endoplasmic reticulum
osa00970 Aminoacyl-tRNA biosynthesis
osa00910 Nitrogen metabolism
Regulated.Type
Down
Up
Sample
CK24vsCK0
OX0vsCK0
OX24vsCK24
OX24vsOX0

Fig. S7

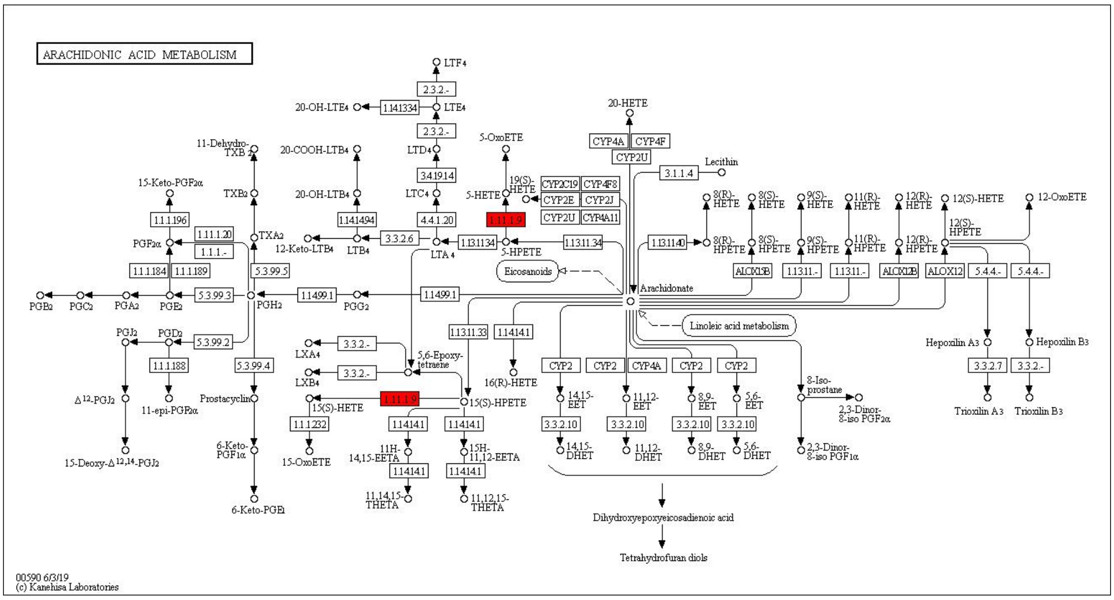
ARACHIDONIC ACID METABOLISM
LTF4
2.3.2.-
20-OH-LTE4
1.14.13.34
LTE4
2.3.2.-
20-HETE
11-Dehydro-TXB2
20-COOH-LTB4
LTD4
5-OxoETE
CYP4A
CYP4F
CYP2U
3.4.19.14
Lecithin
3.1.1.4
15-Keto-PGF2α
TXB2
20-OH-LTB4
LTC4
5-HETE
19(S)-HETE
CYP2C19
CYP4F8
CYP2E
CYP2J
8(R)-HETE
8(S)-HETE
9(S)-HETE
11(R)-HETE
12(R)-HETE
12(S)-HETE
12-OxoETE
1.1.1.196
1.14.14.94
4.4.1.20
1.11.1.9
CYP2U
CYP4A11
12(S)-HPETE
1.11.1.20
TXA2
3.3.2.6
1.13.11.34
1.13.11.34
1.13.11.40
8(R)-HPETE
8(S)-HPETE
9(S)-HPETE
11(R)-HPETE
12(R)-HPETE
PGF2α
1.1.1.-
12-Keto-LTB4
LTB4
LTA4
5-HPETE
Eicosanoids
ALOX15B
1.13.11.-
1.13.11.-
ALOX12B
ALOX12
5.4.4.-
5.4.4.-
1.1.1.184
1.1.1.189
5.3.99.5
Arachidonate
PGB2
PGC2
PGA2
PGE2
5.3.99.3
PGH2
1.11.1.9
PGG2
1.14.99.1
PGJ2
PGD2
5.3.99.2
3.3.2.-
1.13.11.33
1.14.14.1
Linoleic acid metabolism
LXA4
5,6-Epoxy-tetraene
Hepoxilin A3
Hepoxilin B3
1.1.1.188
5.3.99.4
3.3.2.-
16(R)-HETE
CYP2
CYP2
CYP4A
CYP2
CYP2
8-Iso-prostane
LXB4
Δ12-PGJ2
11-epi-PGF2α
Prostacyclin
1.11.1.9
15(S)-HETE
15(S)-HPETE
14,15-EET
11,12-EET
8,9-EET
5,6-EET
2,3-Dinor-8-iso PGF2α
3.3.2.7
3.3.2.-
Trioxilin A3
Trioxilin B3
1.1.1.232
1.14.14.1
1.14.14.1
3.3.2.10
3.3.2.10
3.3.2.10
3.3.2.10
6-Keto-PGF1α
15-OxoETE
11H-14,15-EETA
15H-11,12-EETA
14,15-DHET
11,12-DHET
8,9-DHET
5,6-DHET
2,3-Dinor-8-iso PGF1α
15-Deoxy-Δ12,14-PGJ2
1.14.14.1
1.14.14.1
11,14,15-THETA
11,12,15-THETA
6-Keto-PGE1
Dihydroxyepoxyeicosadienoic acid
Tetrahydrofuran diols
00590 6/3/19
(c) Kanehisa Laboratories

Fig. S8

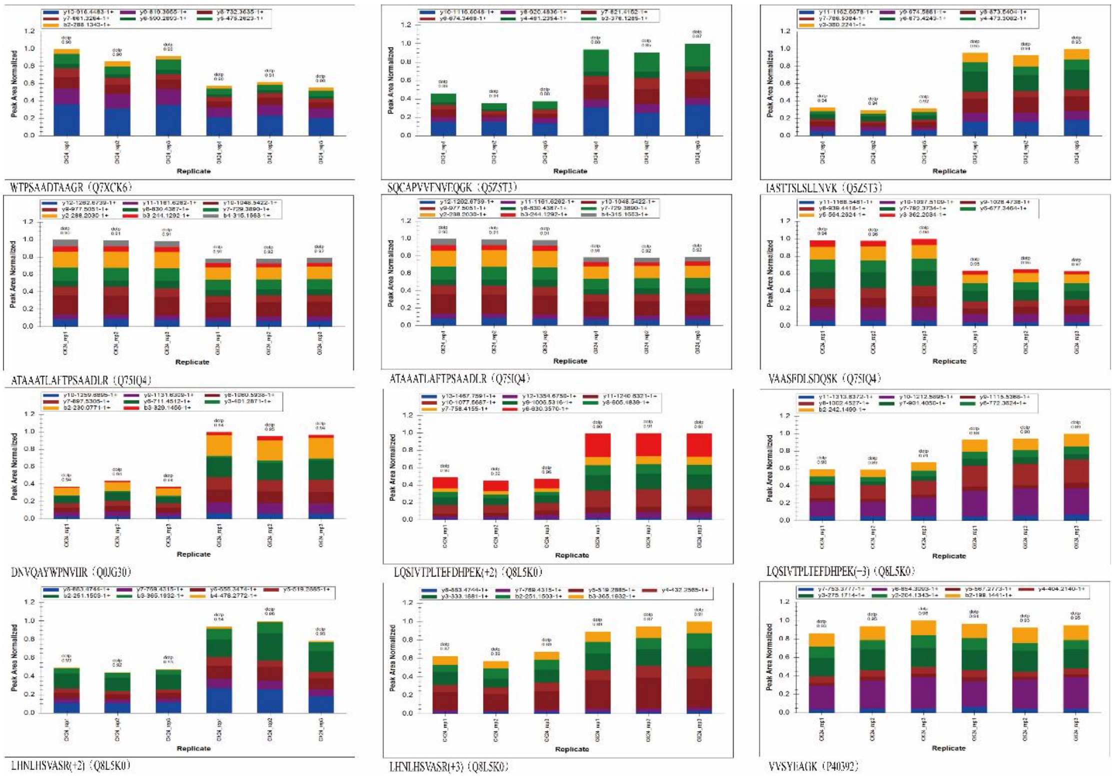
Peak Area Normalized
Replicate
WTPSAADTAAGR (Q7XCK6)
SQCAPVVFNVFQGK (Q5Z5T3)
IASTTSLSLLNVK (Q5Z5T3)
ATAAATLAFTPSAADLR (Q75IQ4)
ATAAATLAFTPSAADLR (Q75IQ4)
VAASFDLSDQSK (Q75IQ4)
DNVQAYWPNVIIR (Q0JG30)
LQSIVTPLTEFDHPEK(+2) (Q8L5K0)
LQSIVTPLTEFDHPEK(+3) (Q8L5K0)
LHNLHSVASR(+2) (Q8L5K0)
LHNLHSVASR(+3) (Q8L5K0)
VVSYEAGK (P40392)

Fig. S9

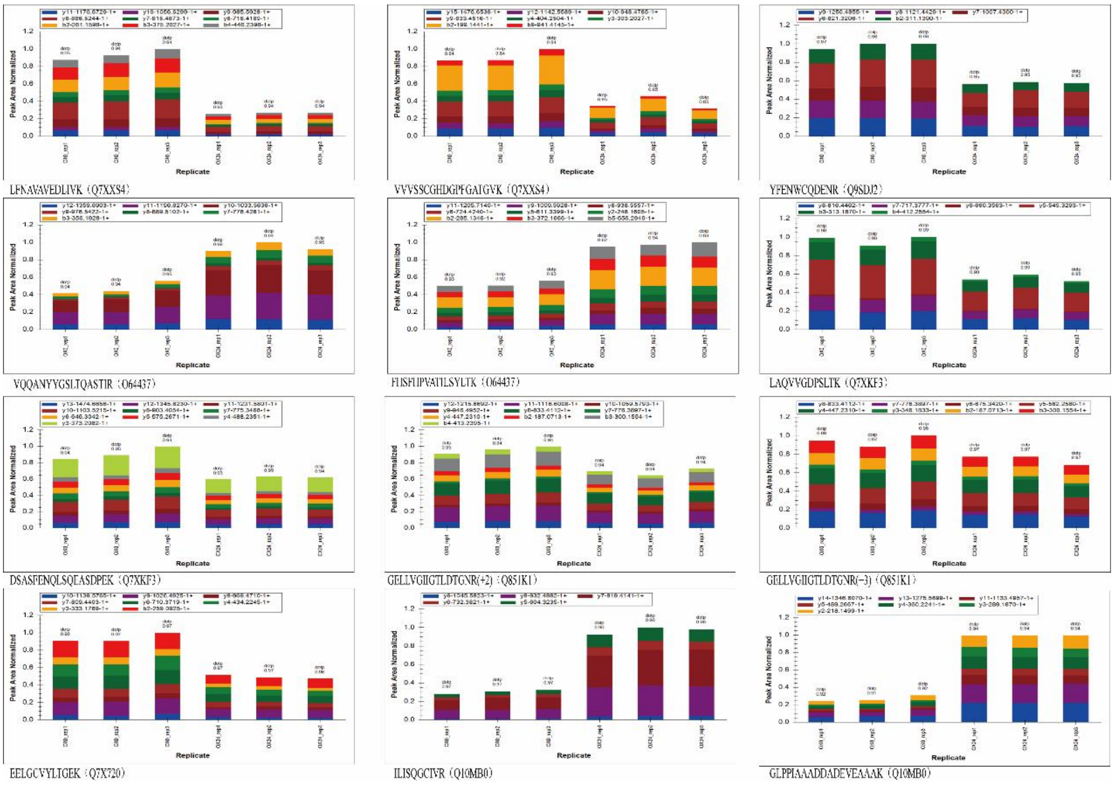
Peak Area Normalized
Replicate
LFNAVAVEDLIVK（Q7XXS4）
VVVSSCGHDGPFGATGVK（Q7XXS4）
YFENWCQDENR（Q9SDJ2）
VQQANYYGSLTQASTIR（O64437）
FIISFIIPVATILSYLTK（O64437）
LAQVVGDPSLTK（Q7XKF3）
DSASFENQLSQLASDPEK（Q7XKF3）
GELLVGIIGTLDTGNR(+2)（Q851K1）
GELLVGIIGTLDTGNR(+3)（Q851K1）
EELGCVYLIGEK（Q7X720）
ILISQGCIVR（Q10MB0）
GLPPIAAADDADEVEAAAK（Q10MB0）

Fig. S10
